# Nonsense-mediated decay controls the reactivation of the oncogenic herpesviruses EBV and KSHV

**DOI:** 10.1371/journal.pbio.3001097

**Published:** 2021-02-17

**Authors:** Michiel van Gent, Adrian Reich, Sadanandan E. Velu, Michaela U. Gack

**Affiliations:** 1 Florida Research and Innovation Center, Cleveland Clinic, Port Saint Lucie, Florida, United States of America; 2 Department of Microbiology, The University of Chicago, Chicago, Illinois, United States of America; 3 Department of Chemistry, University of Alabama Birmingham, Birmingham, Alabama, United States of America; University of Wisconsin-Madison, UNITED STATES

## Abstract

The oncogenic human herpesviruses Epstein–Barr virus (EBV) and Kaposi’s sarcoma-associated herpesvirus (KSHV) are the causative agents of multiple malignancies. A hallmark of herpesviruses is their biphasic life cycle consisting of latent and lytic infection. In this study, we identified that cellular nonsense-mediated decay (NMD), an evolutionarily conserved RNA degradation pathway, critically regulates the latent-to-lytic switch of EBV and KSHV infection. The NMD machinery suppresses EBV and KSHV Rta transactivator expression and promotes maintenance of viral latency by targeting the viral polycistronic transactivator transcripts for degradation through the recognition of features in their 3′ UTRs. Treatment with a small-molecule NMD inhibitor potently induced reactivation in a variety of EBV- and KSHV-infected cell types. In conclusion, our results identify NMD as an important host process that controls oncogenic herpesvirus reactivation, which may be targeted for the therapeutic induction of lytic reactivation and the eradication of tumor cells.

## Introduction

Herpesviruses are large, enveloped DNA viruses that establish widespread persistent infections. The 2 human oncogenic gammaherpesviruses Epstein–Barr virus (EBV) and Kaposi’s sarcoma-associated herpesvirus (KSHV) are carried by a large proportion of the adult population worldwide and pose a significant risk of infection-associated morbidity and mortality, especially in immunocompromised hosts [1–3]. For example, both EBV and KSHV cause a range of malignancies of lymphoid, epithelial, and endothelial origin, and KSHV remains one of the leading causes of death in HIV patients [2,4,5]. Following primary infection, EBV and KSHV typically establish a lifelong latent infection in B cells that is characterized by the near-complete absence of viral gene expression. Occasional reactivation in a small proportion of infected cells, which involves the coordinated expression of the full repertoire of viral lytic genes, leads to the production of viral particles and transmission to new cells and hosts [6–9]. While major improvements have been made in our understanding of the viral factors involved in the latent-to-lytic switch, the molecular details of the host factors that drive viral reactivation remain poorly understood. Notably, latently infected cells are resistant to currently available anti-herpesvirus drugs, which exclusively target the lytic phase of infection; the latent viral reservoirs therefore pose a major obstacle to eliminating persistent EBV and KSHV infection [10,11]. Furthermore, low-level viral reactivation is an important contributor to gammaherpesvirus-associated persistence and tumorigenesis [12,13]. Gaining molecular insight into the factors that modulate latency and reactivation of these viruses is thus of fundamental importance for the development of more effective therapeutic strategies to treat gammaherpesvirus-associated diseases.

Nonsense-mediated decay (NMD) is an evolutionarily conserved cotranslational RNA degradation process that eliminates mRNA transcripts harboring premature termination codons (PTCs) or other, less common, NMD-inducing features [14,15]. Historically, NMD has been known to target faulty mRNA transcripts, which can arise through aberrant splicing or mutagenesis, for degradation to prevent the expression of nonfunctional or dominant-negative proteins that could jeopardize cellular integrity. However, more recently, it has become clear that a significant portion of “intact” cellular transcripts contain NMD-inducing features that allow cells to regulate their expression level and maintain homeostasis in response to environmental changes such as those encountered during development, cellular differentiation, and stress [16,17].

An important prerequisite for PTC recognition by the NMD machinery is the splicing-dependent deposition of exon junction complexes (EJCs), which typically contain the NMD proteins UPF2 and UPF3b, more than 50 to 55 nucleotides (nt) downstream of the PTC [18,19]. The presence of the EJC causes stalling of the ribosome and the translation termination complex at the PTC, which favors recruitment of the key NMD factor UPF1. Subsequent phosphorylation of UPF1 by the serine/threonine kinase SMG1 facilitates an interaction between UPF1 and UPF2/UPF3b. This triggers translational repression and targets the transcript for degradation by various (indirect) nucleolytic pathways through recruitment of additional NMD factors such as the nuclease SMG6 or the SMG5/SMG7 dimer [14]. Alternatively, NMD can be initiated in an EJC-independent manner by the presence of an unusually long (>1 kb) 3′ UTR [17,20]. This process is less well understood and is likely also induced by delayed translation termination that increases the chance of UPF1 recruitment and phosphorylation at the terminating ribosome [14].

Besides its role in maintaining cellular transcriptome integrity and homeostasis, a role for NMD in antiviral immunity to several positive-sense RNA viruses as well as retroviruses in both humans and plants has recently been identified [21–27]. In this study, we show that NMD is an important regulator of reactivation of the oncogenic human DNA viruses EBV and KSHV. Affinity purification of UPF1 combined with RNA sequencing (RNA-seq) analysis identified the polycistronic transcripts encoding the EBV and KSHV Rta transactivators as bona fide NMD targets. We further found that recognition of these transcripts by the NMD machinery relies on specific properties of their long 3′ UTR. Moreover, we show that the small-molecule NMD inhibitory compound NMDI-1 potently induces EBV and KSHV reactivation in various cell types, suggesting that NMD inhibition could provide an unexplored avenue in the treatment of human gammaherpesvirus-associated diseases and malignancies.

## Results

### NMD restricts spontaneous reactivation of the oncogenic herpesviruses EBV and KSHV

**Fig 1:**
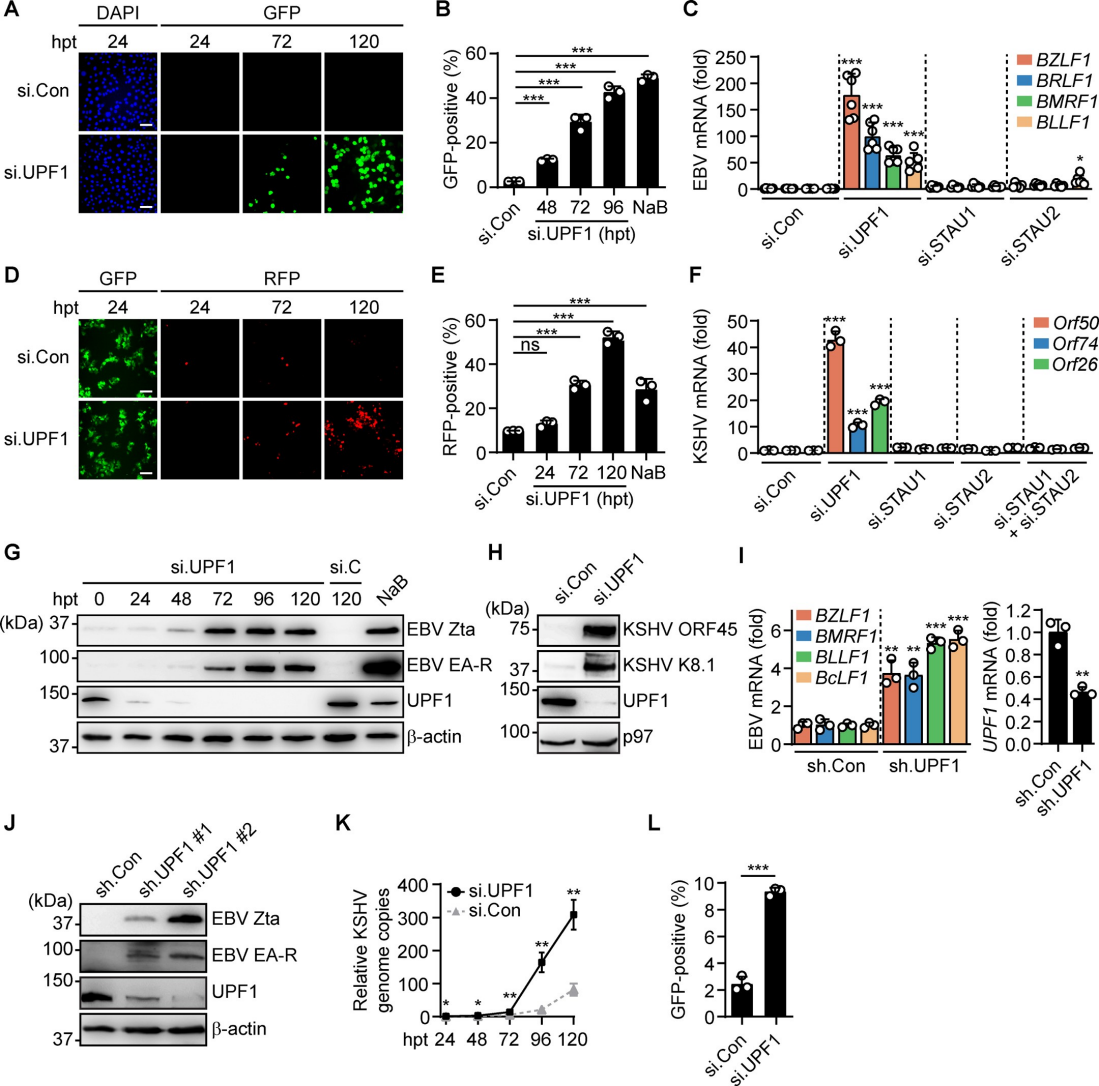
NMD restricts spontaneous reactivation of the oncogenic herpesviruses EBV and KSHV. **(A, B)** Microscopy (A) and flow cytometry (B) analysis of GFP (green) expression as a marker for EBV reactivation in AGS-EBV cells at the indicated hpt with UPF1-specific siRNAs (si.UPF1) or nontargeting control siRNAs (si.Con). Treatment with 2.5 mM sodium butyrate (NaB) for 24 hours served as positive control for reactivation. DAPI, nuclear stain (blue). Scale bar, 50 μm. **(C)** Abundance of lytic EBV transcripts *BZLF1*, *BRLF1*, *BMRF1*, and *BLLF1* in AGS-EBV cells transfected with si.Con or siRNAs targeting UPF1, STAU1, or STAU2 for 96 hours, determined by qRT-PCR and presented as fold expression relative to si.Con. **(D, E)** Microscopy (D) and flow cytometry (E) analysis of RFP (red) expression as a marker for KSHV reactivation in HEK293T.rKSHV219 cells at the indicated hpt with si.UPF1 or si.Con. Constitutively expressed GFP (green) served as a marker for total KSHV^+^ cell number. Scale bar, 50 μm. **(F)** KSHV lytic *Orf50*, *Orf74*, and *Orf26* levels in HEK293T.rKSHV219 cells assessed by qRT-PCR at 96 hpt with siRNAs specific to UPF1, STAU1 and/or STAU2, or si.Con, presented as fold abundance relative to si.Con. **(G)** Immunoblotting analysis of EBV Zta and EA-R as well as cellular UPF1 protein levels in AGS-EBV cells transfected with si.UPF1 or nontargeting control siRNA (si.C) for 0 to 120 hours. β-actin served as loading control. See S1B Fig for quantification of effects. **(H)** Immunoblotting analysis of cellular UPF1 and KSHV ORF45 and K8.1 protein expressions in HEK293T.rKSHV219 cells transfected with si.UPF1 or si.Con for 96 hours. p97 served as loading control. See S1C Fig for quantification of effects. **(I)** qRT-PCR analysis of the indicated lytic EBV genes (left panel) and *UPF1* (right panel) in AKBM cells at 96 hours after lentiviral transduction with an shRNA targeting UPF1 (sh.UPF1) or a nontargeting control shRNA (sh.Con), presented as fold abundance relative to sh.Con. **(J)** Cellular UPF1 and EBV Zta and EA-R protein abundances in AKBM cells at 120 hours post-transduction with 2 individual shRNAs targeting UPF1 (sh.UPF1 #1 and #2) or sh.Con, assessed by immunoblotting with specific antibodies. See S1D Fig for quantification of effects. **(K)** Quantification of relative KSHV genome copies in the supernatant of HEK293T.rKSHV219 cells following transfection with si.UPF1 (black, solid line) or si.Con (gray, dashed line) for 24–120 hours, determined by qPCR with primers specific to *Orf26*. **(L)** Percentage of GFP-positive, EBV-infected HEK293T cells following 30 hours incubation with the supernatant of HEK293T.rKSHV219 cells that were transfected for 96 hours with si.Con or si.UPF1, determined by flow cytometry. Data are representative of at least 2 (B, K, L) or 3 (A, C, D, E, F, G, H, I, J) independent experiments. Pooled data are presented as mean ± SD of at least 3 biological replicates; ns, *p* > 0.05, * *p* ≤ 0.05, ** *p* ≤ 0.01, *** *p* ≤ 0.001; 1-way ANOVA for B, C, E, F; 2-sided Student *t* test for I, K, L. The underlying numerical data can be found in S1 Data; original immunoblots can be found in S1 Raw Images. See also S1 and S2 Figs. EBV, Epstein–Barr virus; GFP, green fluorescent protein; hpt, hours post-transfection; KSHV, Kaposi’s sarcoma-associated herpesvirus; NMD, nonsense-mediated decay; qPCR, quantitative PCR; qRT-PCR, quantitative reverse transcription PCR; RFP, red fluorescent protein; shRNA, short hairpin RNA; siRNA, small interfering RNA.

The oncogenic human herpesviruses EBV and KSHV encode many spliced, polycistronic transcripts that typically display the primary features of canonical NMD targets, such as a stop codon upstream of an EJC and/or a long 3′ UTR downstream of the proximal ORF [28–31]. We therefore hypothesized that NMD regulates maintenance of viral latency and/or lytic reactivation by controlling the expression of certain gammaherpesvirus transcripts. To test this, we started by determining the effect of small interfering RNA (siRNA)-mediated depletion of the critical NMD component UPF1 on spontaneous EBV reactivation in the human gastric carcinoma cell line AGS-EBV, which harbors recombinant EBV Akata-BX1 that encodes green fluorescent protein (GFP) under control of the lytic BXLF1 promoter [32]. Fluorescence microscopy and flow cytometry analyses of AGS-EBV cells showed that UPF1 silencing markedly increased the proportion of cells expressing GFP, a measure of EBV reactivation, to a similar extent as treatment with sodium butyrate (NaB), a known potent inducer of EBV reactivation that served as a positive control (**Fig 1A and 1B**). Furthermore, evaluation of EBV lytic gene expression by quantitative reverse transcription PCR (qRT-PCR) showed robust up-regulation of EBV lytic genes *BZLF1*, *BRLF1*, *BMRF1*, and *BLLF1* following UPF1 depletion (**Figs 1C and S1A**). Notably, silencing of Staufen 1 or 2 (STAU1 or STAU2), critical components of the STAU-mediated RNA degradation pathway that is related to NMD and also critically relies on UPF1 [33], did not substantially induce lytic gene expression (**Figs 1C and S1A**). In parallel, we tested the effect of NMD inhibition on KSHV reactivation in HEK293T.rKSHV219 cells, which are latently infected with recombinant KSHV.219 that constitutively expresses GFP and encodes red fluorescent protein (RFP) under control of the viral lytic polyadenylated nuclear (PAN) RNA promoter [34]. UPF1 silencing in these cells resulted in a robust increase in the proportion of cells expressing RFP, a marker for KSHV reactivation (**Fig 1D and 1E**), and efficiently induced expression of the KSHV lytic genes *Orf50*, *Orf74*, and *Orf26* (**Fig 1F**). Silencing of STAU1 or STAU2 alone, or in combination, did not induce KSHV lytic gene expression (**Fig 1F**). We also observed that UPF1 depletion resulted in strongly enhanced expression of the EBV lytic proteins Zta and EA-R in AGS-EBV cells as well as KSHV lytic proteins ORF45 and K8.1 in HEK293T.rKSHV219 cells (**Figs 1G and 1H and S1B and S1C**). Next, we asked whether robust reactivation of EBV upon UPF1 silencing was also observed in other relevant cell types, such as the human Akata EBV^+^ Burkitt lymphoma AKBM cells [35]. We found that short hairpin RNA (shRNA)-mediated depletion of endogenous UPF1 resulted in significant up-regulation of EBV lytic genes *BZLF1*, *BMRF1*, *BLLF1*, and *BcLF1* as well as an increased abundance of the lytic proteins Zta and EA-R (**Figs 1I and 1J and S1D**).

Importantly, depletion of the other NMD components UPF2, UPF3b, SMG1, SMG5, SMG6, or SMG7 also induced EBV lytic gene and protein expression (although with different potencies) in AGS-EBV cells, as well as KSHV lytic gene expression in HEK293T.rKSHV219 and iSLK.rKSHV219 cells, demonstrating that viral reactivation is induced by general interference with NMD activity rather than UPF1 silencing specifically (**S2A–S2D Fig**).

EBV and KSHV reactivation is a highly regulated process that consists of sequential expression of viral immediate-early transactivators, early genes, and late genes that ultimately results in the production of infectious viral particles [6,7,36]. However, under some conditions, reactivation is abortive, characterized by limited viral lytic gene expression and the absence of viral particle production [37,38]. To determine whether NMD inhibition induces bona fide, productive EBV and KSHV reactivation, we performed whole viral transcriptome analysis by RNA-seq and observed that UPF1 depletion enhanced transcriptional activity along the entire viral genome for both EBV and KSHV, similar to NaB treatment (**S3A and S3B Fig**). We also observed that UPF1 silencing in HEK293T.rKSHV219 cells resulted in enhanced production of KSHV particles released into the supernatant as compared to control cells (**Fig 1K**) and, correspondingly, a greater infectivity when this supernatant was used to infect naïve HEK293T cells (**Fig 1L**).

Taken together, these results demonstrate that inhibition of NMD robustly induces productive EBV and KSHV reactivation in various latently infected cell types, resulting in the enhanced production of infectious virus particles.

### Gammaherpesvirus transcripts are targeted by the NMD machinery

**Fig 2:**
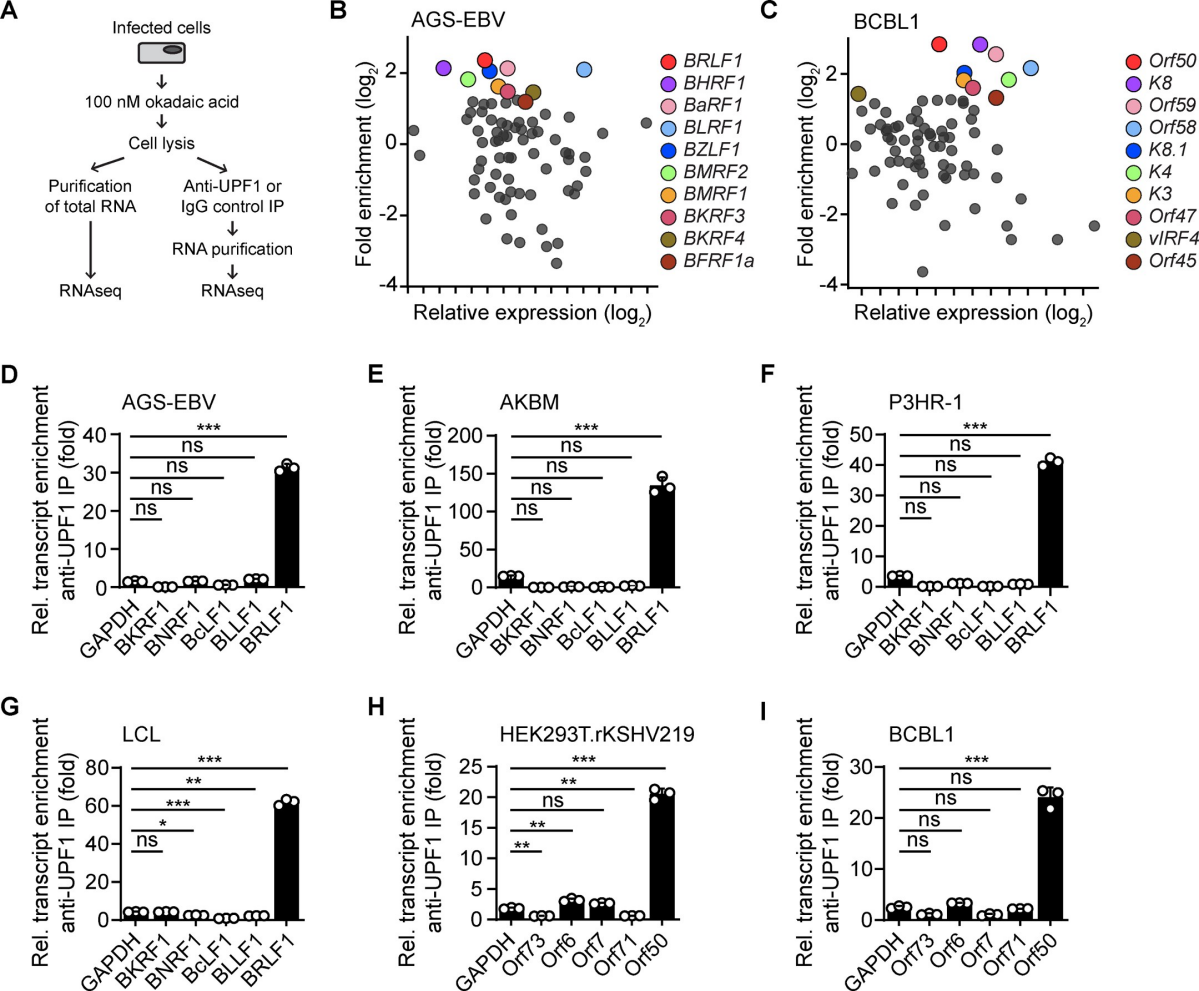
Gammaherpesvirus transcripts are targeted by the NMD machinery. **(A)** Schematic of the UPF1 affinity purification and RNA-seq (RIP-seq) approach used to identify viral NMD targets. **(B, C)** EBV and KSHV transcript enrichment with UPF1 following UPF1 IP from AGS-EBV (B) or BCBL1 (C) cell extracts, respectively, determined by RNA-seq analysis and presented as fold enrichment in the UPF1 IP relative to the IgG control IP (log_2_ values; y-axis) versus relative abundance in the total input RNA (log_2_ values; x-axis). Labels in plot indicate the 10 viral transcripts with the greatest enrichment. **(D–I)** qRT-PCR analysis of the enrichment of the indicated viral transcripts with UPF1 following UPF1 IP from extracts of EBV^+^ AGS-EBV (D), AKBM (E), P3HR-1 (F), or LCL (G) cells, as well as KSHV^+^ HEK293T.rKSHV219 (H) or BCBL1 (I) cells, presented as fold enrichment in the UPF1 IP relative to the IgG control IP (normalized to *18S*). *GAPDH*, a cellular transcript that is not sensitive to NMD, served as control. Data are representative of at least 2 independent experiments and presented as mean ± SD of 3 technical replicates; ns, *p* > 0.05, ** *p* ≤ 0.01, *** *p* ≤ 0.001; 1-way ANOVA. The underlying numerical data can be found in S1 Data; RNA-seq data have been deposited under NCBI BioProject accession number PRJNA677887. See also S4 and S5 Figs. EBV, Epstein–Barr virus; GFP, green fluorescent protein; IP, immunoprecipitation; KSHV, Kaposi’s sarcoma-associated herpesvirus; LCL, lymphoblastoid cell line; NMD, nonsense-mediated decay; qRT-PCR, quantitative reverse transcription PCR; RIP-seq, RNA immunoprecipitation sequencing; RNA-seq, RNA sequencing.

Although many cellular NMD-targeted transcripts have been identified, it remains largely obscure which viral transcripts are degraded by NMD, in particular those expressed by DNA viruses. Thus, we next sought to identify the EBV and KSHV transcripts that are targeted by NMD using a UPF1-RNA immunoprecipitation sequencing (RIP-seq) approach that has been commonly used to identify cellular NMD targets [39,40]. Latently infected AGS-EBV cells were pretreated with the protein phosphatase 2a inhibitor okadaic acid to inhibit dephosphorylation of UPF1 and promote the interaction between (phospho-)UPF1 and NMD-targeted transcripts. Cell extracts were prepared, and immunoprecipitations were performed using anti-UPF1, or Immunoglobulin G (IgG) as a negative control, followed by the purification of UPF1- (or IgG-) associated RNAs and RNA-seq analysis (**Fig 2A**). In untreated AGS-EBV cells, we found 10 viral transcripts to be significantly enriched with UPF1 (relative to the IgG control IP), of which the EBV transactivator-encoding *BRLF1* transcript was most highly enriched with UPF1 (**Fig 2B**). *BZLF1*, which is encoded by the polycistronic *BRLF1* transactivator transcript of EBV as well as by a separate monocistronic transcript, was also among the top 5 enriched genes. Of note, all except one of the significantly enriched viral transcripts were part of EBV gene clusters that share a common polyadenylation site and give rise to polycistronic transcripts, including the BaRF1-BMRF1-BMRF2 and BKRF3-4 clusters (**S4A Fig**). Importantly, the enrichment of viral transcripts with UPF1 did not correlate with their overall abundance in the infected cell (**Fig 2B**, x-axis). In parallel, this RIP-seq assay was also performed with extracts from AGS-EBV cells treated with NaB for 24 hours to induce lytic reactivation and thereby increase the abundance of lytic transcripts. Under these conditions, we found *BRLF1* to be the third most highly enriched viral transcript (**S4B Fig**). Furthermore, we performed the same analysis following precipitation of phosphorylated UPF1 (p-UPF1) using a phospho-specific antibody from untreated and NaB-treated AGS-EBV cells. While this resulted in fewer significantly enriched viral transcripts overall, *BRLF1* was still the top viral transcript enriched with p-UPF1 in untreated cells and the fourth highest enriched transcript in NaB-treated cells (**S4C and S4D Fig**).

Similarly, we assessed the enrichment of KSHV transcripts with UPF1 in KSHV^+^ primary effusion lymphoma (PEL) BCBL1 and HEK293T.rKSHV219 cells. Analogous to our observations for EBV, we found that the polycistronic KSHV transactivator-encoding *Orf50* transcript was the viral transcript most highly enriched with UPF1 in BCBL1 cells and among the top 10 enriched viral transcripts in HEK293T.rKSHV219 cells (**Figs 2C and S4E**). Furthermore, K8 and K8.1, which are encoded by the polycistronic *Orf50* transcript, and also smaller transcripts similar to *BZLF1* for EBV, were among the top enriched genes with UPF1 in both KSHV^+^ cell lines tested. Similar to our data for EBV transcripts, all except one of the highly enriched KSHV transcripts were derived from gene clusters with a shared polyadenylation site (**S4F Fig**).

To corroborate the association of UPF1 with the EBV and KSHV transactivator transcripts detected in our RIP-seq approach, we analyzed the UPF1-associated RNAs in several latently infected cells by qRT-PCR. We validated this assay by confirming the successful precipitation of phosphorylated (“activated”) UPF1 by immunoblotting with anti-p-UPF1 (**S5A Fig**) and the enrichment of the known cellular NMD-sensitive transcripts *MAP3K14*, *GADD45B*, and *PDRG1* [41], but not the NMD-insensitive transcripts *GAPDH*, *RPL32*, or *HPRT1*, with UPF1 by qRT-PCR (**S5B–S5G Fig**). In EBV^+^ AGS-EBV, Burkitt lymphoma AKBM and P3HR-1, as well as LCL cells, we observed a striking enrichment of *BRLF1* transcripts with UPF1 relative to the IgG control IP (**Fig 2D–2G**). Other transcripts, such as cellular *GAPDH* or EBV *BKRF1*, *BNRF1*, *BcLF1*, and *BLLF1* that were not enriched in our RIP-seq analysis, were not found to be significantly enriched by qRT-PCR either. We also observed robust and specific enrichment of *Orf50* transcripts with UPF1 in KSHV^+^ HEK293T.rKSHV219 and BCBL1 cells, corroborating the RIP-seq results (**Fig 2H and 2I**).

Taken together, these results identify that the EBV *BRLF1* and KSHV *Orf50* transactivator-encoding transcripts are highly associated with UPF1 in several EBV- or KSHV-infected cell types.

### NMD controls the abundance of the polycistronic EBV and KSHV transactivator transcripts

**Fig 3:**
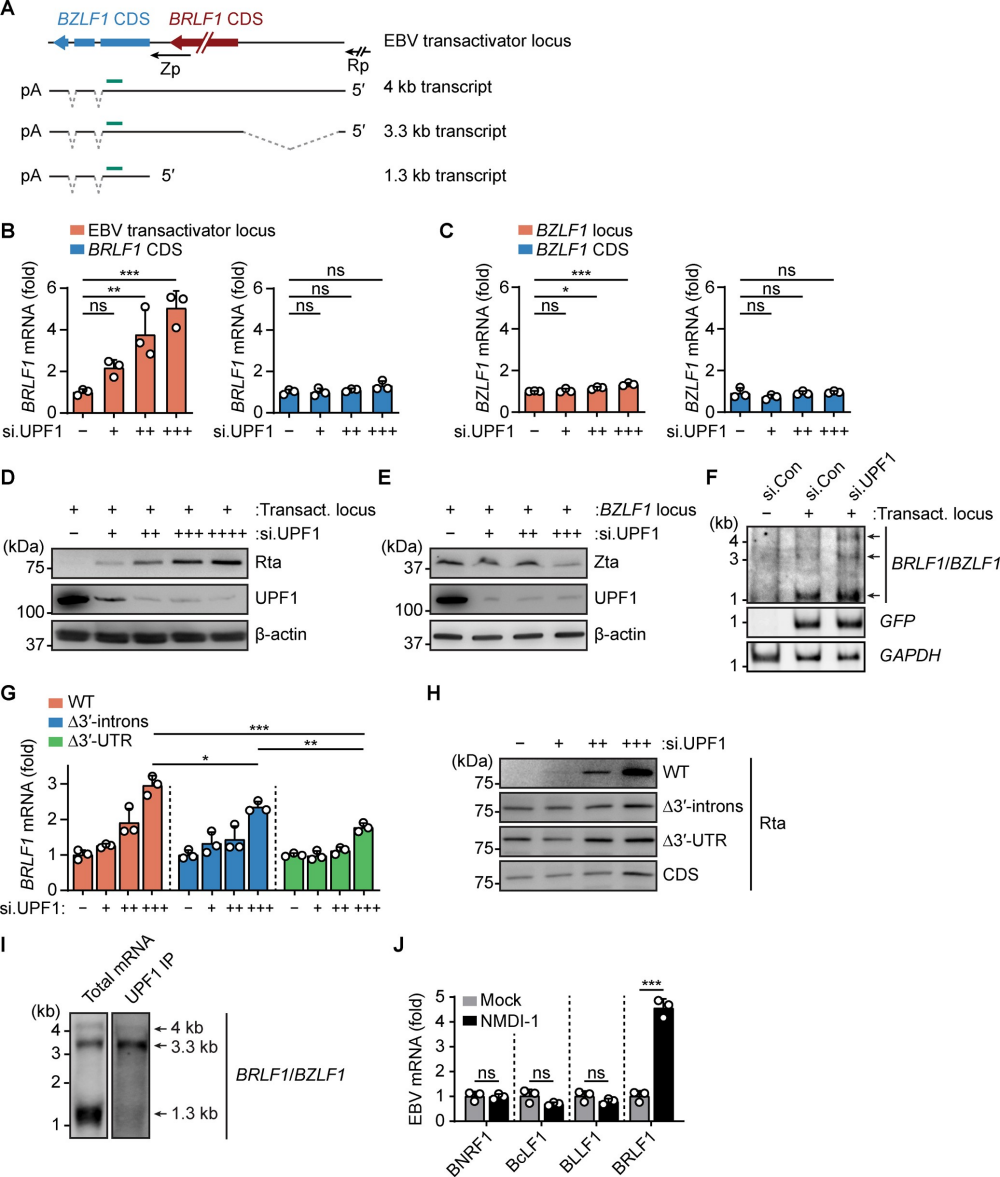
NMD controls the abundance of the polycistronic EBV transactivator transcripts and Rta protein expression. **(A)** Schematic of the EBV transactivator locus and the 3 transcripts expressed from this locus. Dashed lines mark spliced introns; CDS, coding sequence; Rp, EBV *BRLF1* promoter; Zp, EBV *BZLF1* promoter; pA, 3′-polyadenylation site; green bar, location of the northern blotting probe used in F and I. **(B)** qRT-PCR analysis of *BRLF1* transcripts in HEK293T cells transfected with 0, 20, 40, or 60 nM UPF1-targeting siRNA for 24 hours, followed by transfection of a plasmid encoding the complete EBV transactivator locus (left panel, orange) or the *BRLF1* CDS only (right panel, blue), together with pcDNA3-nlsGFP (internal control) for 48 hours. Data were normalized to *GFP* transcript levels to control for differences in transfection efficiency and presented as fold abundance relative to cells without si.UPF1 (-). **(C)** qRT-PCR analysis of *BZLF1* transcripts in HEK293T cells transfected with increasing amounts of si.UPF1 for 24 hours as in (B), followed by transfection for 48 hours with a plasmid encoding the EBV *BZLF1* locus (left panel, orange) or the *BZLF1* CDS (right panel, blue), together with pcDNA3-nlsGFP (internal control). Data were normalized and presented as in (B). **(D)** Immunoblotting analysis of Rta and cellular UPF1 protein in HEK293T cells transfected with 0, 20, 40, 60, or 80 nM si.UPF1 for 24 hours, followed by transfection of a plasmid encoding the complete EBV transactivator locus for 48 hours. β-actin served as loading control. See S6E Fig for quantification of effects. **(E)** Immunoblotting analysis of Zta and cellular UPF1 protein in HEK293T cells transfected with 0, 20, 40, or 60 nM si.UPF1 for 24 hours, followed by transfection with a plasmid encoding the EBV *BZLF1* locus for 48 hours. β-actin served as loading control. See S6F Fig for quantification of effects. **(F)** Northern blotting analysis of *BRLF1* and *BZLF1* transcripts using the probe indicated in (A), in HEK293T cells transfected with 80 nM si.Con or si.UPF1 for 24 hours, followed by transfection for 48 hours with an empty control vector (-) or a plasmid encoding the complete EBV transactivator locus (+), together with pcDNA3-nlsGFP. Arrows indicate the 4-, 3.3-, and 1.3-kb transcripts derived from the transactivator locus. *GFP* served as internal control for transfection efficiency. *GAPDH* served as loading control. **(G)** qRT-PCR analysis of *BRLF1* transcripts in HEK293T cells transfected with 0, 20, 40, or 60 nM si.UPF1 for 24 hours, followed by transfection of a plasmid encoding the WT EBV transactivator locus (orange) or mutant plasmids lacking either the 2 *BZLF1* introns (Δ3′-introns, blue) or the entire 3′ UTR (Δ3′ UTR, green), together with pcDNA3-nlsGFP, for 48 hours. Data are presented as fold expression relative to the sample without si.UPF1 (-) after normalization to *GFP* transcript abundance. **(H)** Immunoblotting analysis of Rta protein in HEK293T cells transfected as in (G). See S6G Fig for quantification of effects. **(I)** Northern blotting analysis of transactivator transcripts in total mRNA or UPF1-associated RNA following UPF1 IP from AKBM cells, using the probe indicated in (A). Arrows mark the 3 transcripts derived from the transactivator locus. **(J)** qRT-PCR analysis of the indicated EBV transcripts in AGS-EBV cells treated with 5 ng/mL TPA without (Mock) or with 25 μM NMDI-1 for 24 hours. Data are presented as fold abundance relative to the mock-treated sample. Data are representative of at least 2 (D, E, J) or 3 (B, C, F, G, H, I) independent experiments. Pooled data are presented as mean ± SD of 3 biological replicates; ns, *p* > 0.05, * *p* ≤ 0.05, ** *p* ≤ 0.01, *** *p* ≤ 0.001; 1-way ANOVA for B, C, and G; 2-sided Student *t* test for J. The underlying numerical data can be found in S1 Data; original immunoblots and northern blots can be found in S1 Raw Images. See also S6 Fig. EBV, Epstein–Barr virus; IP, immunoprecipitation; NMD, nonsense-mediated decay; qRT-PCR, quantitative reverse transcription PCR; WT, wild-type.

Expression of the viral Rta transactivator proteins encoded by the EBV *BRLF1* and KSHV *Orf50* transcripts is required and sufficient to induce the lytic reactivation cascade of these viruses [42,43]. Our observations that these transcripts associate with UPF1 led us to hypothesize that NMD-mediated degradation minimizes transactivator expression levels in latently infected cells to prevent gammaherpesvirus reactivation and, conversely, that NMD inhibition increases transactivator abundance resulting in viral reactivation. To test this hypothesis, we focused on the EBV transactivator locus, which is comprised of the *BRLF1* and *BZLF1* genes and gives rise to at least 3 different transcripts of 4 kb, 3.3 kb, and 1.3 kb in size that encode the EBV transactivator proteins Rta and/or Zta (**Fig 3A**). To test the effect of NMD inhibition on EBV transactivator transcript and protein abundance in the absence of other viral proteins, the entire EBV transactivator locus consisting of *BRLF1* and *BZLF1* under control of the endogenous Rp promoter region (nt −1 to −987 relative to the transcription start site (TSS) for *BRLF1* [44]) was expressed from a eukaryotic expression vector in naïve HEK293T cells. Cotransfected GFP-encoding plasmid served as an internal control to normalize for general differences in transfection and/or transcription efficiency between samples. We observed that NMD inhibition by either siRNA-mediated UPF1 silencing or overexpression of the dominant-negative UPF1-R843C mutant [45] led to a dose-dependent increase in *BRLF*1 transcript abundance (**Figs 3B and S6A**, left panels). In contrast, NMD inhibition did not significantly affect *BRLF1* transcript abundance when expressed from a control plasmid that solely encoded the *BRLF1* coding sequence (CDS) under control of the same viral Rp promoter (**Figs 3B and S6A**, right panels). Expression of a construct encoding the *BZLF1* locus under control of the endogenous Zp promoter (nt −1 to −552), or a corresponding control plasmid encoding only the *BZLF1* CDS, showed that monocistronic *BZLF1* transcript levels were minimally affected by UPF1 silencing or UPF1-R843C overexpression (**Figs 3C and S6B**). Analogously, we tested whether expression of the polycistronic KSHV *Orf50* transcripts was suppressed by NMD by generating an expression plasmid encoding the entire KSHV transactivator locus consisting of *Orf50*, *K8*, and *K8*.*1* under control of the endogenous *Orf50* promoter (nt −1 to −3,079 relative to the *Orf50* TSS [46]) (**S6C Fig**). Similar to our results for EBV *BRLF1*, expression of this plasmid in UPF1-silenced cells resulted in a dose-dependent increase in *Orf50* transcript abundance, whereas expression from an *Orf50* CDS-only control plasmid was not affected (**S6D Fig**).

Next, we tested whether the increase in EBV *BRLF1* transcript levels upon NMD inhibition resulted in enhanced Rta protein abundance. Indeed, UPF1 depletion followed by transfection of the full-length transactivator locus expression plasmid caused an increase in EBV Rta abundance that correlated with the amount of transfected si.UPF1 (**Figs 3D and S6E**). Conversely, in line with our observation that the monocistronic *BZLF1* transcript was not degraded by NMD, transfection of the plasmid encoding only the *BZLF1* locus showed that Zta levels were not increased upon silencing of UPF1 (**Figs 3E and S6F**).

To corroborate these results, we depleted endogenous UPF1 from HEK293T cells using siRNA followed by transfection of the full-length transactivator plasmid and then evaluated transcript abundance by northern blotting using a probe that recognizes all 3 EBV transactivator transcripts (see **Fig 3A**). In line with our earlier observations, the larger 3.3- and 4-kb polycistronic *BRLF1* transcripts became more abundant following UPF1 silencing, whereas the abundance of the 1.3-kb *BZLF1*-encoding transcript was similar in control siRNA-transfected cells and UPF1-depleted cells (**Fig 3F**). Taken together, these results show that NMD specifically induced the degradation of the viral polycistronic transactivator transcripts, which limits Rta gene and protein expression.

### NMD targets the transactivator transcripts by recognizing features in their 3′ UTR

Our finding that the polycistronic *BRLF1* transcripts, but not the *BRLF1* CDS-only or the monocistronic *BZLF1* transcripts, are sensitive to NMD suggests that the *BRLF1* transcripts contain specific properties that facilitate recognition by the NMD machinery. The polycistronic *BRLF1* transcripts display 2 primary NMD-inducing features: They possess a long 3′ UTR as well as 2 splice sites more than 55 nt downstream of the *BRLF1* stop codon that can facilitate EJC deposition (see **Fig 3A**). To determine which of these feature(s) sensitize(s) the *BRLF1* transcripts to NMD-mediated degradation, we generated 2 mutant *BRLF1* constructs lacking either the two 3′ introns that reside within the *BZLF1* coding region (Δ3′-introns) or the entire 3′ UTR downstream of the *BRLF1* stop codon (Δ3′ UTR). The transcripts derived from the plasmid lacking the two 3′ introns displayed reduced up-regulation upon UPF1 depletion as compared to transcripts derived from the wild-type (WT) transactivator locus, while deletion of the entire 3′ UTR further reduced the effect of UPF1 silencing on transcript levels (**Fig 3G**). In accord, whereas increased Rta protein abundance was observed upon UPF1 depletion when expressed from the full-length construct, Rta protein levels were only slightly affected by UPF1 depletion when expressed from plasmids lacking either the 3′ introns or the entire 3′ UTR, similarly to Rta expression from the CDS-only plasmid (**Figs 3H and S6G**). These results suggest that primarily splicing-dependent EJC deposition in the 3′ UTR, but also the long 3′ UTR itself, are responsible for the sensitization of *BRLF1* transcripts to NMD-mediated degradation.

### NMD suppresses BRLF1 expression in virus-infected cells

Next, we sought to corroborate these findings in virus-infected cells by determining which *BRLF1* transcripts associate with UPF1 in AGS-EBV cells using northern blotting. In line with our previous results, whereas all 3 major transcripts were identified in the total input mRNA pool, primarily the 3.3 kb and, to a much lesser extent, the 4-kb *BRLF1*-encoding transcripts were found to be associated with UPF1. In contrast, the 1.3-kb monocistronic *BZLF1*-encoding transcript was not enriched with UPF1 (**Fig 3I**).

We next asked whether the association of *BRLF1* transcripts with UPF1 led to their degradation in virus-infected cells. Since *BRLF1* transcript up-regulation upon NMD inhibition would, through Rta transactivation activity, result in the induction of global lytic gene expression that would prevent us from evaluating a direct effect of NMD on transcript abundance, we treated AGS-EBV cells with TPA to induce equal *BRLF1* and lytic gene expression in all samples; the abundance of viral lytic transcripts was then analyzed in the presence or absence of the small-molecule NMD inhibitor NMDI-1 [47]. In these experimental settings, NMD inhibition by NMDI-1 treatment resulted in a significant increase in *BRLF1* transcript abundance compared to the mock-treated control, whereas the levels for other EBV transcripts that were not enriched in our original RIP-seq screens, such as *BNRF1*, *BcLF1*, and *BLLF1*, were not significantly affected by NMDI-1 treatment (**Fig 3J**). This strengthens the notion that *BRLF1* transcripts are a direct target of the NMD machinery in EBV-infected cells, whereas other viral lytic transcripts such as *BLLF1* and *BcLF1* are not directly targeted by the NMD machinery, and their up-regulation in non-TPA treated cells (see **Fig 1C and 1I**) is very likely a consequence of the transactivation activity of up-regulated Rta.

Together, these results indicate that NMD targets and degrades *BRLF1* transcripts during authentic EBV infection.

### Small-molecule NMD inhibitor NMDI-1 is a potent inducer of EBV and KSHV reactivation

EBV and KSHV are each responsible for a significant number of cancer cases each year. The currently available anti-herpesvirus drugs, such as ganciclovir, rely on expression of the viral kinases for their activation and therefore exclusively target reactivated cells [11]. While lytic infection is increasingly appreciated to contribute to EBV- and KSHV-associated malignancies, the far majority of virus-positive tumor cells are latently infected and thus insensitive to the currently available antiviral drugs [12]. For this reason, there is a strong interest in the development of therapeutic strategies to induce reactivation and sensitize tumor cells to antiviral drugs. Since we identified the cellular NMD pathway as an important regulator of EBV and KSHV reactivation, we next sought to investigate the therapeutic potential of small-molecule NMD inhibitory compounds by examining their ability to induce gammaherpesvirus reactivation. We focused on NMDI-1, a compound that inhibits NMD by blocking the interaction between SMG5 and UPF1 [47].

**Fig 4:**
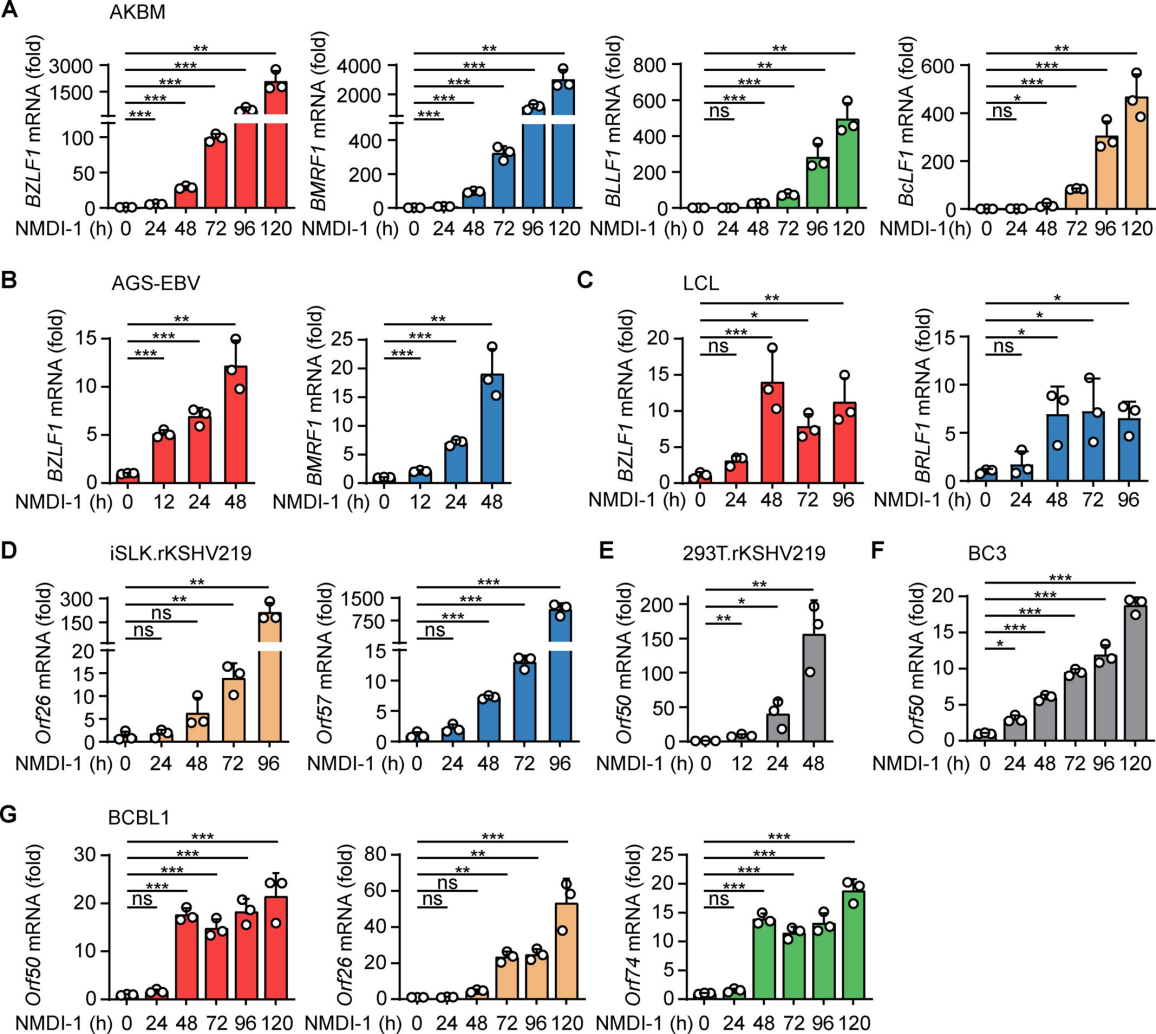
Small-molecule NMD inhibitor NMDI-1 is a potent inducer of EBV and KSHV reactivation. **(A–C)** qRT-PCR analysis of EBV *BZLF1*, *BRLF1*, *BMRF1*, *BLLF1b*, and/or *BcLF1* transcripts in EBV^+^ AKBM (A), AGS-EBV (B), or LCL (C) cells treated with 25 μM NMDI-1 for 12 to 120 hours, presented as fold expression relative to mock-treated samples. **(D–G)** qRT-PCR analysis of *Orf26*, *Orf50*, *Orf57*, or *Orf74* transcripts in KSHV^+^ iSLK.rKSHV219 (D), HEK293T.rKSHV219 (E), BC3 (F), and BCBL1 (G) cells treated with 25 μM NMDI-1 for the indicated hours, presented as fold expression relative to mock-treated samples. Data are representative of at least 2 (A, C) or 3 (B, D, E, F, G) independent experiments and presented as mean ± SD of 3 biological replicates; ns, *p* > 0.05, * *p* ≤ 0.05, ** *p* ≤ 0.01, *** *p* ≤ 0.001. The underlying numerical data can be found in S1 Data. EBV, Epstein–Barr virus; KSHV, Kaposi’s sarcoma-associated herpesvirus; LCL, lymphoblastoid cell line; NMD, nonsense-mediated decay; qRT-PCR, quantitative reverse transcription PCR.

To test the effect of NMDI-1 on viral reactivation, we treated EBV^+^ AKBM cells with 25 μM NMDI-1 and assessed up-regulation of the lytic genes *BZLF1*, *BMRF1*, *BLLF1*, and *BcLF1* by qRT-PCR as a measure for viral reactivation (**Fig 4A**). We observed that NMDI-1 treatment resulted in a striking induction of EBV lytic gene expression in AKBM cells. Similarly, a potent induction of lytic gene expression upon treatment with NMDI-1 was observed in AGS-EBV cells as well as in EBV-transformed LCL cells derived from healthy donor primary B cells (**Fig 4B and 4C**). Of note, NMDI-1 at concentrations between 5 and 50 μM did not or only minimally affect cell viability (**S7A and S7B Fig**). Similarly to the effects seen on EBV reactivation, treatment of KSHV^+^ iSLK.rKSHV219, HEK293T.rKSHV219, and BCBL1 or BC3 PEL cells with NMDI-1 induced potent expression of the KSHV lytic transcripts *Orf50*, *Orf26*, *Orf57*, and/or *Orf74* (**Fig 4D–4G**).

In conclusion, our results demonstrate that the small-molecule NMD-inhibitor NMDI-1 effectively induces EBV and KSHV reactivation in a variety of cell types.

## Discussion

NMD plays a well-documented role in regulating the abundance of a large variety of cellular transcripts; however, our knowledge of the interplay between NMD and viral infection, in particular infection with DNA viruses, remains rudimentary. Along these lines, although recent reports have revealed a contribution of NMD to RNA virus infections, only few bona fide viral NMD targets have been identified [21–27]. In this study, we show that NMD targets the spliced, polycistronic EBV and KSHV transactivator-encoding transcripts for degradation through the recognition of NMD-inducing features in their 3′ UTRs; this ultimately keeps the abundance of the EBV and KSHV Rta proteins to a minimum, thereby suppressing virus reactivation. Our findings thus identify NMD as a key regulator of oncogenic DNA virus infection.

The biphasic life cycle consisting of latency establishment in long-lived cells and the occasional reactivation that results in production of viral progeny is a hallmark of herpesvirus infection. Given the central role of the Rta transactivator proteins of EBV and KSHV in initiating lytic reactivation, it is not surprising that their expression is extensively regulated. The cotranslational regulation of Rta mRNA stability by NMD that we identified here provides an extra layer of control in addition to the epigenetic, transcriptional, and posttranscriptional regulatory mechanisms that have been reported previously [37,48–50]. Mechanistically, our results suggest that NMD prevents viral reactivation by degrading transactivator transcripts that are produced at low levels in latently infected cells. The strong lytic reactivation that we observed upon inhibition of NMD, to a similar extent as treatment with potent chemical inducers such as sodium butyrate, underscores the importance of NMD in preserving EBV and KSHV latency. Future studies will need to determine the exact interplay and temporal sequence of the various regulatory mechanisms that modulate EBV and KSHV reactivation.

Our results also indicated that the NMD-mediated degradation of EBV BRLF1-encoding transcripts depends on the presence of introns as well as the long 3′ UTR. Our results are consistent with the findings by Zhao and colleagues who recently reported that the 3′ UTR of KSHV *Orf50* transcripts is recognized by the NMD machinery in an intron-dependent manner, and that, consequently, silencing of the NMD components UPF1 or UPF3X resulted in de-repression of KSHV reactivation [51]. The conservation of these NMD-inducing features in the transactivator locus of EBV and KSHV may suggest that these viruses have adopted NMD-mediated regulation of viral gene expression and reactivation as an integral part of their life cycle. Besides the transactivator-encoding transcripts that we identified as NMD targets, gammaherpesvirus genomes also encode many other spliced and/or polycistronic transcripts that often display features known to induce NMD-mediated degradation [28–31]. Several of these transcripts were found to be associated with UPF1 in our RIP-seq analysis. Future studies will need to investigate whether other viral transcripts are targeted by the NMD machinery, which would further expand the role of NMD in regulating EBV and KSHV gene expression. Furthermore, the Gene Set Enrichment Analysis (GSEA) of UPF1-associated cellular transcripts showed that 14 gene sets were significantly enriched with UPF1 or phospho-UPF1 in EBV-infected cells, suggesting that NMD may also modulate the activity of several cellular processes during infection (**S8 Fig**). It will be interesting to determine the extent to which NMD-mediated control of these pathways regulates EBV and KSHV reactivation, especially because some of these pathways have previously been implicated in the regulation of EBV and/or KSHV reactivation; these include the unfolded protein response, whose regulation by NMD was recently shown to affect KSHV reactivation [51] and the MYC pathway that was recently reported to suppress EBV reactivation [52]. In view of this, it seems plausible that EBV and KSHV have evolved mechanisms to manipulate NMD-mediated transcript degradation. While to date these functions have not been identified for herpesviruses, several positive-sense RNA viruses as well as retroviruses have recently been shown to compromise NMD activity by interfering with the function of specific NMD factors; furthermore, certain cellular transcripts escape NMD by encoding specific features that inhibit NMD-mediated degradation [22,23,25,27,53–55]. Taken together, we anticipate future studies to reveal a more intricate and dynamic interplay between gammaherpesvirus infection and the host NMD pathway.

The balance between latent and lytic infection is a significant determinant in EBV- and KSHV-associated tumorigenesis [12,13]. Given the important role of NMD in regulating the latent-to-lytic switch, it will be important to determine how changes in NMD activity by host regulatory mechanisms or environmental triggers affect gammaherpesvirus replication and oncogenesis. For example, reported variations in intrinsic NMD activity between cells may contribute to gammaherpesvirus tissue and cell tropism, and although we observed robust EBV reactivation by NMD inhibition in all cell types tested, there might still be differences in NMD-mediated control of Rta expression and/or cellular pathways in epithelial versus lymphoid cells [56]. Finally, besides cell type–specific differences, variations in NMD efficiency between individuals, which have been shown to affect the outcome of certain genetic diseases, may affect susceptibility to EBV- and KSHV-associated diseases [57].

Since the majority of cells in EBV- and KSHV-associated cancers are latently infected, therapeutic induction of lytic reactivation can be used to specifically sensitize tumor cells to antiviral drugs and to activate cytotoxic T-lymphocyte responses that are typically directed against lytic antigens [12,48,58,59]. In this study, we observed a strong induction of EBV and KSHV reactivation by the small-molecule NMD-inhibitor NMDI-1 in a variety of cell types, even those that are notoriously refractory to reactivation. Concentrations of NMDI-1 below those used in our study have successfully been used in in vivo studies without apparent toxicity [60,61]. Moreover, it was recently reported that modest NMD inhibition does not have an appreciable negative impact on overall health in mice [62]. Together, this suggests that NMD inhibition may be a potential strategy to therapeutically induce viral reactivation in the treatment of EBV- and KSHV-associated malignancies.

In conclusion, our study identifies the cellular NMD pathway as a prominent regulator of EBV and KSHV reactivation, providing novel insight into the intricate virus–host interactions that play a fundamental role in viral disease progression.

## Methods

### Cell culture

HEK293T cells (ATCC) were maintained in Dulbecco’s Modified Eagle Medium (DMEM, Gibco, United States of America) supplemented with 10% (v/v) heat-inactivated fetal bovine serum (FBS), 2 mM GlutaMAX (Gibco), and 1% (v/v) penicillin-streptomycin (Pen-Strep, Gibco) under standard tissue culture conditions. HEK293T.rKSHV219 and iSLK.rKSHV219 cells (kindly provided by D. Ganem, University of California) were maintained in DMEM supplemented with 10% (v/v) FBS, 2 mM GlutaMAX, 1% (v/v) Pen-Strep, and 2 μg/mL puromycin (Sigma, USA). AGS (ATCC) and AGS-EBV cells (kindly provided by N. Raab-Traub, University of North Carolina, Chapel Hill [32]) were cultured in F-12 nutrient mixture (Gibco) supplemented with 10% FBS, 2 mM GlutaMAX, 1% (v/v) Pen-Strep, and 500 μg/mL G418 (Sigma). BJAB, P3HR-1 (kindly provided by B. Gewurz, Harvard Medical School), and AKBM cells (kindly provided by M. Ressing, Leiden University Medical Center, the Netherlands [35]) were cultured in Roswell Park Memorial Institute (RPMI) medium (Gibco) supplemented with 10% (v/v) FBS, 2 mM GlutaMAX, 1% (v/v) Pen-Strep, and 0.3 mg/mL hygromycin B. The PEL cells BC3 and BCBL1 were cultured in RPMI supplemented with 20% (v/v) FBS, 2 mM GlutaMAX, and 1% (v/v) Pen-Strep. LCL cells were prepared by incubating human healthy donor–derived CD19^+^ B cells (iXCells) with EBV^+^ supernatant from sodium butyrate-treated AGS-EBV cells. The LCL phenotype of the outgrowing cells was confirmed by flow cytometry analysis of CD19 expression and immunoblotting analysis of EBV EBNA1 expression. LCLs were maintained in RPMI supplemented with 10% (v/v) FBS, 2 mM GlutaMAX, and 1% (v/v) Pen-Strep.

### Plasmids and transfections

pcDNA3-nlsGFP was kindly provided by M. Ressing (Leiden University Medical Center, the Netherlands). UPF1 was subcloned with an N-terminal HA tag from pCMV6-UPF1-MYC-DDK (Origene, NM_001297549) into a dual promoter lentiviral vector (BIC-PGK-Zeo-T2a- mAmetrine [63]; kindly provided by R.J. Lebbink, University Medical Center Utrecht, the Netherlands) under control of the human EF1A promoter. To generate the dominant-negative UPF1-R843C mutant, a gBlock (IDT) encoding the required mutation was used to replace the corresponding region of UPF1 using the SbfI and PmlI restriction sites and the Gibson Assembly method.

All EBV and KSHV transactivator-encoding plasmids were generated using Gibson assembly in a pCMV6 vector backbone from which the CMV-IE promoter was removed. All fragments used for cloning, as indicated below, were either generated by PCR or ordered as gBlocks (IDT) using sequences from the EBV Akata genome (GenBank accession number KC207813.1) or KSHV JSC-1 BAC16 genome (GenBank accession number GQ994935) as templates. For the plasmid containing the entire EBV transactivator locus, a fragment corresponding to nt 94,567 to 89,497 of the EBV Akata genome, encompassing the entire region from the start of the Rp promoter until the polyadenylation site, was introduced into the pCMV6 backbone. The mutant “Δ3′-introns” plasmid was generated by deleting the 2 introns corresponding to nt 89,797 to 89,713 and 90,053 to 89,903 of the EBV genome. To generate the Δ3′ UTR plasmid, the entire sequence between the *BRLF1* stop codon and the polyadenylation site was deleted. To obtain the *BRLF1* CDS-only plasmid, the sequence downstream of the Rp promoter in the full-length plasmid was replaced with the *BRLF1* CDS only, corresponding to nt 92,582 to 90,765 of the EBV Akata genome. To generate the plasmid encoding the *BZLF1* locus, a fragment corresponding to nt 91,145 to 89,497 of the EBV genome, encompassing the region from the start of the Zp promoter until the polyadenylation site, was introduced into the pCMV6 backbone. For the *BZLF1* CDS-only plasmid, the 3 *BZLF1* exons corresponding to nt 90,554 to 90,054, nt 89,902 to 89,798, and nt 89,712 to 89,581 of the EBV genome were joined together downstream of the Zp promoter. To generate the plasmid containing the entire KSHV transactivator locus, a fragment corresponding to nt 68,333 to 76,595 of the BAC16 genome, encompassing the entire region from the start of the *Orf50* promoter until the polyadenylation site, was introduced into the pCMV6 backbone lacking the CMV-IE promoter. To obtain the *Orf50* CDS-only control plasmid, the 2 Rta-encoding *Orf50* exons corresponding to nt 71,412 to 71,429 and 72,388 to 74,445 in the KSHV genome were joined together downstream of the *Orf50* promoter sequence. The sequence of all constructs was verified by Sanger sequencing.

To assess transactivator transcript and protein expression levels, HEK293T cells were transfected with increasing amounts (0 nM, 20 nM, 40 nM, 60 nM, or 80 nM) of UPF1-targeting siRNAs, supplemented with nontargeting control siRNAs to 80 nM, using Lipofectamine RNAiMAX transfection reagent in 12-well plates following the manufacturer’s instructions. After 24 hours, plasmid transfections were performed using 1.6 μg plasmid DNA (100 ng plasmid of interest, 100 ng pcDNA3-nlsGFP, and 1,400 ng empty pCMV6 control vector) per well using Lipofectamine2000 (Life Technologies, USA) or polyethylenimine (PEI; Polysciences, USA) according to the manufacturer’s instructions. Forty-eight hours after DNA transfection, the cells were harvested for qRT-PCR or immunoblotting analysis.

### siRNA-mediated silencing

Transient knockdown of endogenous genes was achieved by transfection of the indicated target cells with 80 nM of gene-specific siRNAs using Lipofectamine RNAiMAX transfection reagent (Life Technologies) in 12-well plates according to the manufacturer’s instructions. siRNAs targeting the following genes were purchased as siGENOME SMARTpools from Dharmacon (USA): UPF1 (M-011763-01), STAU1 (M-011894-01), STAU2 (M-006873-00), UPF2 (M-012993-01), UPF3a (M-012872-00), UPF3b (M-012871-00), SMG1 (M-005033-01), SMG5 (M-014023-00), SMG6 (M-017845-01), SMG7 (M-021305-01), and a nontargeting control (D-001206-14). After 24 to 120 hours, the cells were harvested for further analysis as indicated. Knockdown efficiency was assessed in each experiment by measuring transcript or protein abundance by qRT-PCR or immunoblotting.

### Lentiviral transduction of AKBM cells

Standard lentivirus production methods were used to generate in HEK293T cells third generation lentiviral particles for 2 shRNAs targeting UPF1 (Sigma TRC human genome wide shRNA library; pLKO.1 backbone; #1 5′-GCATCTTATTCTGGGTAATAA-3′ and #2 5′- GCCTACCAGTACCAGAACATA-3′) as well as a nontargeting control shRNA. Transduction of AKBM cells was performed by adding 1 mL of lentivirus preparation to 0.5 million AKBM cells in a 12-well plate. Forty-eight hours later, cells were selected using 0.4 μg/mL puromycin, and samples were harvested at the indicated times for analysis of viral gene expression by qRT-PCR or protein expression by immunoblotting analysis.

### Quantitative reverse transcription PCR (qRT-PCR)

Total RNA was extracted from cells using the E.Z.N.A. HP Total RNA Isolation Kit (Omega Bio-tek, USA) according to the manufacturer’s instructions. Reverse transcription and qRT-PCR were performed using equal amounts (50 to 500 ng) of the purified RNA and the SuperScript III Platinum One-Step qRT-PCR kit with ROX (Invitrogen, USA) on a 7500 Fast Real-Time PCR Machine (Applied Biosystems, USA). Premixed master mixes of TaqMan primers and probes for the detection of human transcripts were purchased from Applied Biosystems (18S) or IDT (GAPDH, UPF1, UPF2, UPF3a, UPF3b, SMG1, SMG5, SMG6, SMG7, STAU1, STAU2, GADD45B, PDRG1, RPL32, HPRT1, and MAP3K14). To detect viral genes, the following custom PrimeTime primer/probe mixes were ordered from IDT: EBV *BZLF1* (forward primer 5′-GGAAACCACTACAGCCAGAA-3′, reverse primer 5′-AGCAGCCACCTCACGGTA-3′, probe 5′-ACAAGAATCGGGTGGCTTCCAGAA-3′), *BRLF1* (forward primer 5′-ACCTCACTACACAAACAGACG-3′, reverse primer 5′-TGTTGAGGACGTTGCAGTAG-3′, probe 5′-AGCCTCAGAAAGTCTTCCAAGCCATC-3′), *BMRF1* (forward primer 5′-CAACACCGCACTGGAGAG-3′, reverse primer 5′-GCCTGCTTCACTTTCTTGG-3′, probe 5′-AGGAAAAGGACATCGTCGGAGGC-3′), *BLLF1* (forward primer 5′-TGGGATGTAGACAAGTTACGCCT-3′, reverse primer 5′-TGCTGACCCTTCTGCTGCT-3′, probe 5′-TCATGGCGGACTGCGCCTT-3′), *BcLF1* (forward primer 5′-TGCATGGCGGTCATTCC-3′, reverse primer 5′-CATGGGCAAATACGCGG-3′, probe 5′-ATGTCTTCCTTCCCTCGTTTCAATCAG-3′), *BKRF1* (forward primer 5′-TACAGGACCTGGAAATGGCC-3′, reverse primer 5′-TCTTTGAGGTCCACTGCC-3′, probe 5′-AGGAAGACTCATCTGGACCAGAAGGC-3′), *BNRF1* (forward primer 5′- GGAGTTTCCCCCGATTCAAG-3′, reverse primer 5′-TCCATGCTCTCGTCCACATCT-3′, probe 5′-AGGGCGCAAGTTCTCCGGTACCC-3′); and KSHV *Orf50* (forward primer 5′- CACAAAAATGGCGCAAGATGA-3′, reverse primer 5′-TGGTAGAGTTGGGCCTTCAGTT-3′, probe 5′-AGAAGCTTCGGCGGTCCTG-3′), *Orf74* (forward primer 5′- GTTCCCCTGATATACTCCTGC-3′, reverse primer 5′-GGACATGAAAGACTGCCTGAG-3′, probe 5′-AGGATGTACGGTCTCTTCCAAAGCC-3′), *Orf26* (forward primer 5′-GCTAGCAGTGCTACCCCCACT-3′, reverse primer 5′-GTCAAATCCGTTGGATTCG-3′, probe 5′-AGCCGAAAGGATTCCACCATTGTGC-3′), *Orf6* (forward primer 5′-TTCTGTGACCTCTTTGACACC-3′, reverse primer 5′-GCATTGCTCTGGCTATCCT-3′, probe 5′-AAACATCCCTCCTATGGCAGCGTC-3′), *Orf7* (forward primer 5′- GAACACGTAGAGATCCTGACAC-3′, reverse primer 5′-ACATTTGGAGGACTGGGAAATA-3′, probe 5′-TCTACAAACTTATCACGGGCCCGC-3′), *Orf71* (forward primer 5′-CTTACACTGGGTGTACTGTATGG-3′, reverse primer 5′- GCTGTAGGTCTACTCTTGACAAA-3′, probe 5′-CCACTGACGTGGATGCCCTAATGT-3′), and *Orf73* (forward primer 5′-CCCTTAACGAGAGGAAGTTGTAG-3′, reverse primer 5′- TTCCTTCGCGGTTGTAGATG-3′, probe 5′-AAGATGTGACCTTGGCGATGACCT-3′). Abundance of target genes was calculated by normalizing for cellular *18S* or *GAPDH* using the comparative CT (ΔΔCt) method. Data were displayed as mean fold expression (+/− SD) compared to control samples, which were set to 1.

For quantification of KSHV viral genome copies, cell supernatant was treated with DNase to eliminate free, non-capsid-associated DNA, followed by purification of viral DNA using the QIAamp MinElute Virus Vacuum Kit (Qiagen, USA). The purified DNA was subsequently used as input for the qPCR reactions using *Orf26*-specific primers.

### Immunoblotting analysis

Cell lysates were prepared in NP-40 buffer (150 mM NaCl, 1% (v/v) NP-40, 50 mM HEPES pH 7.4, and protease inhibitor cocktail (Sigma)) or RIPA buffer (25 mM Tris HCl pH 7.6, 150 mM NaCl, 1% (v/v) NP-40, 1% (wt/v) sodium deoxycholate, 0.1% (wt/v) SDS, and protease inhibitor cocktail (Sigma)). Cell debris was pelleted by centrifugation at >13,000 x *g* for 20 minutes at 4°C, and proteins were denatured by incubation at 95°C for 2 minutes in 1x Laemmli Sample Buffer (Bio-Rad, USA). Samples were resolved by Bis-Tris-PAGE using the Mini-Protean Tetra Cell system (Bio-Rad) and 1x MOPS-SDS running buffer (Alfa Aesar, USA), followed by transfer onto polyvinylidene difluoride (PVDF) membranes (Bio-Rad) using a Novex semidry transfer cell (Invitrogen) or Mini Trans-Blot Cell wet transfer system (Bio-Rad). After blocking with 5% (wt/v) nonfat dry milk in PBS-Tween 20 for 1 hour at room temperature (RT), membranes were probed with primary antibodies for either 1 hour at RT or at 4°C overnight. The primary antibodies used were anti-Zta (1:500, sc-53904, Santa Cruz Biotechnology, USA), anti-Rta (1:250, 8C12, provided by R. Feederle, Helmholtz Zentrum München, Germany), and anti-EA-R (1:250, sc-56979, Santa Cruz Biotechnology) to detect EBV proteins; anti-ORF45 (1:200, sc-53883, Santa Cruz Biotechnology) and anti-K8.1 (1:200, sc-65446, Santa Cruz Biotechnology) to detect KSHV proteins; and anti-UPF1 (1:2,000, D15G6, Cell Signaling Technology, USA), anti-phosphoUPF1 (Ser1127, 1:1,000, 07–1016, Millipore Sigma, USA), anti-p97 (1:2,000, 612183, BD Biosciences, USA), and anti-β-actin (1:10,000, AC-15, Sigma) to detect cellular proteins. Next, membranes were incubated with goat anti-mouse or goat anti-rabbit horseradish peroxidase (HRP)-conjugated secondary antibodies (1:2,000, #7076S, and #7074S, Cell Signaling Technology) for 1 hour at RT. Protein bands were visualized using the enhanced SuperSignal West Pico or Femto chemiluminescence reagent (Thermo Fisher Scientific, USA) and were detected using a LAS Imagequant 4000 luminescent imaging system (GE Life Sciences, USA). Densitometric quantification was performed using ImageQuant TL software (GE Life Sciences).

### Northern blotting analysis

Total mRNA was purified from cell pellets using the Dynabeads mRNA DIRECT Purification kit (Thermo Fisher Scientific) according to the manufacturer’s instructions. RNA was denatured at 70°C for 5 minutes in 1x RNA loading dye (Thermo Fisher Scientific) and resolved on a 1.5% agarose gel using a Mini-Protean Tetra Cell system (Bio-Rad) in 1x TBE buffer (Invitrogen). After electrophoresis, the RNA was transferred onto a BrightStar Plus positively charged Nylon membrane (Invitrogen) using a Criterion cell (Bio-Rad) in 0.5 x TBE buffer and UV-crosslinked to the membrane at 300 mJ/cm^2^. The membrane was hybridized with biotinylated probes purchased from IDT, recognizing the EBV transactivator transcripts (5′-CATAAGCTTGATAAGCATTCTCAGGAGCAGGCTGAGGGGC-3′), *GFP* (5′- TCGGCGCGGGTCTTGTAGTTGCCGTCGTCCTTGAAGAAGA-3′), or *GAPDH* (5′-TGGTGCAGGAGGCATTGCTGATGATCTTGAGGCTGTTG-3′), in ULTRAhyb-Oligo Hybridization Buffer (Invitrogen) at 42°C overnight, followed by incubation with a streptavidin-alkaline phosphatase conjugate for detection (Invitrogen). Imaging was performed using CDP-Star luminescent substrate (Life Technologies), and bands were detected using a LAS Imagequant 4000 luminescent imaging system (GE Life Sciences).

### Large-scale UPF1 immunoprecipitation

Approximately 25 × 10^6^ cells per sample were seeded into 15-cm dishes and either left untreated or treated with 2.5 mM sodium butyrate (NaB) for 24 hours as indicated. Next, the cells were treated with 100 nM PP2a inhibitor okadaic acid (Cell Signaling Technology) for 3 hours, after which lysates were prepared in NP40 lysis buffer (50 mM HEPES, pH 7.4, 150 mM KCl, 1 mM Na_3_VO_4_, 0.5% (v/v) NP-40, and 0.5 mM Dithiothreitol, supplemented with protease inhibitor (Sigma)) and incubated rotating at 4°C for 30 minutes. The lysates were cleared by centrifugation at 13,000 × *g* for 20 minutes at 4°C. Dynabeads Protein A (Invitrogen) were precoupled with anti-UPF1 (D15G6, Cell Signaling Technology), anti-phosphoUPF1 (Ser1127, Millipore Sigma), or normal rabbit-IgG control (Millipore Sigma) antibodies overnight and mixed with the cleared lysates followed by incubation at 4°C for 4 hours with constant agitation. Precipitates were washed 3 times with NP-40 lysis buffer and twice with high-salt NP40 lysis buffer (containing 300 mM KCl), followed by protein digestion with proteinase K (NEB, USA) for 30 minutes at 55°C and purification of precipitated RNA using phenol/chloroform/isoamylalcohol (Sigma) according to the manufacturer’s instructions.

### RNA-seq analysis

RNA samples were submitted to the University of Chicago Genomics Facility for library preparation and sequencing on a HiSeq4000 instrument using 50-base pair single-end reading (Illumina, USA). Two (for EBV) or 1 (for KSHV) sets of at least 3 pooled independent experiments were separately processed and sequenced. The sequencing data were uploaded to the Galaxy web platform, and the public server at usegalaxy.org was used for analysis [64]. Raw sequence reads were quality trimmed using TRIM Galore! (Galaxy Version 0.6.3) and aligned to the human genome (Gencode GRCh38.p12 v31) as well as the EBV Akata genome (GenBank accession number KC207813.1) for AGS-EBV and AKBM samples, the KSHV JSC-1 BAC16 genome (GenBank accession number GQ994935.1) for HEK293T.rKSHV219 samples, or the KSHV GK18 genome (GenBank accession number NC_009333.1) for BCBL1 samples, using HISAT2 (Galaxy Version 2.1.0) [65]. FeatureCounts (Galaxy Version 1.6.4, [66]) was used to calculate transcript abundance, and significantly enriched genes were determined using DESeq2 (Galaxy Version 2.11.40.6, [67]). Coverage at individual genome positions for the whole transcriptome analysis was calculated using SAMtools mpileup [68]. Graphs were generated using GraphPad Prism software. For the GSEA, transcripts enriched in the phospho-UPF1 and UPF1 IP samples from untreated and NaB-treated AGS-EBV cells were analyzed using GSEA version 4.1.0 against the Molecular Signatures Database (MSigDB; version 7.2) and compared to those of the IgG control IP samples. Significant gene sets were identified for each of the 4 comparisons as having a *p*-value < 0.05 and false discovery rate < 0.25.

### Fluorescence microscopy and flow cytometry

AGS-EBV and HEK293T.rKSHV219 cells were seeded into 12-well plates and transfected with UPF1-specific or nontargeting control siRNAs for 24 to 120 hours as indicated. As a positive control for reactivation, cells were treated with 2.5 mM NaB for 24 hours. For microscopy, cells were incubated with Hoechst nucleic acid stain (1:2,000, Invitrogen) in PBS for 5 minutes at RT, after which DAPI, as well as GFP or RFP were imaged using a fluorescence microscope (Omano, USA). For flow cytometry analysis of GFP and RFP expression, cells were fixed in 4% paraformaldehyde in PBS and analyzed on an LSRFortessa (BD Biosciences). For the determination of the percentage of apoptotic and necrotic cells, cells were incubated with 7-AAD Viability Staining Solutions (BioLegend, USA) and FITC-Annexin V (BioLegend) for 15 minutes at RT following the manufacturer’s instructions, followed by analysis on a BD FACSAriaII (BD Biosciences). Data were analyzed using the Flowjo software (BD Biosciences).

### NMDI-1 treatment

A stock concentration of the NMD inhibitor NMDI-1 [47] was maintained at 10 mM in DMSO. To determine the effect of NMDI-1 treatment on EBV and KSHV reactivation, the indicated cells were seeded into 12-well plates. The next day, the cells were treated with 25 μM of NMDI-1, or the equivalent amount of DMSO as mock treatment. The cells were harvested at the indicated times for analysis of EBV and KSHV lytic gene expression by qRT-PCR.

### Statistical analysis

All pooled data were presented as means ± SD of at least 3 biological replicates and analyzed using GraphPad Prism software. One-way ANOVA with Dunnett’s multiple comparisons or a 2-tailed Student *t* test was used to test for statistical significance as indicated in the figure legends.

## Supporting information

S1 Fig Representative knockdown efficiency of UPF1, STAU1, and STAU2 as well as densitometric quantification of immunoblots presented in Fig 1. **(A)** Representative qRT-PCR analysis of UPF1, STAU1, and STAU2 knockdown efficiency in AGS-EBV cells transfected with the indicated siRNAs for 96 hours, displayed as fold expression relative to si.Con-transfected cells. **(B–D)** Densitometric quantification of the relative signal intensities in 2 or 3 independent biological replicates of the representative immunoblots presented in Fig 1G (B), 1H (C), and 1J (D). The underlying numerical data can be found in S1 Data. EBV, Epstein–Barr virus; qRT-PCR, quantitative reverse transcription PCR; siRNA, small interfering RNA; STAU, Staufen.
(TIF)

S2 Fig Silencing of NMD components induces EBV and KSHV reactivation. **(A)** Immunoblotting analysis of EBV proteins Zta and EA-R in AGS-EBV cells transfected for 96 hours with siRNAs targeting the indicated NMD factors, or a nontargeting control siRNA (si.Con). β-actin served as loading control. Treatment with 2.5 mM sodium butyrate (NaB) for 24 hours served as positive control for reactivation. **(B)** qRT-PCR analysis of EBV lytic genes *BZLF1*, *BMRF1*, and *BcLF1* (left panel) and silencing efficiency of the respective NMD components (right panel) in AGS-EBV cells treated as in (A), presented as fold expression relative to si.Con. **(C)** qRT-PCR analysis of KSHV *Orf50* and *Orf26* transcripts (left panel) and knockdown efficiency of the respective NMD components (right panel) in HEK293T.rKSHV219 cells transfected with siRNAs targeting the indicated NMD genes for 72 hours, presented as fold expression relative to si.Con. **(D)** qRT-PCR analysis of KSHV lytic *Orf26* and *Orf74* transcripts (left panel) and knockdown efficiency of the respective NMD components (right panel) in iSLK.rKSHV219 cells transfected with the indicated siRNAs for 96 hours, presented as fold expression relative to si.Con. Of note, SMG1 could not be efficiently silenced in HEK293T.rKSHV219 or iSLK.rKSHV219 cells and therefore was not tested. Data are presented as mean ± SD of at least 3 biological replicates; * *p* ≤ 0.05, ** *p* ≤ 0.01, *** *p* ≤ 0.001. The underlying numerical data can be found in S1 Data; original immunoblots can be found in S1 Raw Images. EBV, Epstein–Barr virus; KSHV, Kaposi’s sarcoma-associated herpesvirus; NMD, nonsense-mediated decay; qRT-PCR, quantitative reverse transcription PCR; siRNA, small interfering RNA.
(TIF)

S3 Fig EBV and KSHV transcriptome analysis. **(A)** Whole genome RNA-seq coverage plots (log_2_ scale) of RNA-seq reads mapping to the forward (orange) or reverse (blue) strands of the EBV genome. AGS-EBV cells were transfected for 96 hours with UPF1-specific siRNAs (si.UPF1) or nontargeting control siRNAs (si.Con) with or without treatment with 2.5 mM NaB for the last 24 hours, followed by RNA purification and whole transcriptome RNA-seq analysis. Plots are aligned to a schematic representation of the EBV genome depicting annotated ORFs on the forward (orange) and reverse (blue) strands. Lower panel displays the fold induction of mapped reads of the si.UPF1 and NaB-treated samples relative to si.Con sample (log_2_ scale). **(B)** Whole genome RNA-seq coverage plots (log_2_ scale) of RNA-seq reads mapping to the forward (orange) or reverse (blue) strands of the KSHV genome, derived from HEK293T.rKSHV219 cell extracts treated and displayed as in (A). The RNA-seq data have been deposited under NCBI BioProject accession number PRJNA677887. EBV, Epstein–Barr virus; KSHV, Kaposi’s sarcoma-associated herpesvirus; RNA-seq, RNA sequencing.
(TIF)

S4 Fig RIP-seq analysis of EBV and KSHV transcripts associated with endogenous UPF1 or phospho-UPF1. **(A)** Overview of the 10 EBV transcripts presented in Fig 2B that were found to be significantly enriched with endogenous UPF1 in AGS-EBV cells indicating whether they are (blue circle) or are not (red circle) encoded in a viral gene cluster with a shared polyadenylation (poly(A)) site and/or derived from an intron-containing locus. **(B)** Enrichment of UPF1-associated EBV transcripts following UPF1 IP from AGS-EBV cells treated with 2.5 mM NaB for 24 hours, as determined by RNA-seq analysis and presented as fold enrichment in the UPF1 IP relative to the IgG control IP (log_2_ values; y-axis) versus relative abundance in the total input RNA (log_2_ values; x-axis). Labels in plot indicate the 10 viral transcripts with the greatest enrichment. **(C, D**) Enrichment of UPF1-associated EBV transcripts following phospho-UPF1 IP from untreated AGS-EBV cells (C) or AGS-EBV cells treated with 2.5 mM NaB for 24 hours (D), determined by RNA-seq and presented as in (B). Labels in plots indicate the significantly enriched EBV transcripts. **(E)** KSHV transcript enrichment with UPF1 following UPF1 IP from HEK293T.rKSHV219 cell extracts, determined by RNA-seq and presented as in (B). Labels in plot indicate the 10 viral transcripts with the greatest enrichment. **(F)** Overview of the 10 KSHV transcripts presented in Fig 2C that were found to be highly enriched with endogenous UPF1 in BCBL1 cells indicating whether they are (blue circle) or are not (red circle) encoded in viral gene cluster with a shared polyadenylation (poly(A)) site and/or derived from an intron-containing locus. The RNA-seq data have been deposited under NCBI BioProject accession number PRJNA677887. EBV, Epstein–Barr virus; IP, immunoprecipitation; KSHV, Kaposi’s sarcoma-associated herpesvirus; RIP-seq, RNA immunoprecipitation sequencing; RNA-seq, RNA sequencing.
(TIF)

S5 Fig Validation of the UPF1 IP assay to identify NMD-targeted transcripts. **(A)** Representative immunoblotting analysis showing UPF1 phosphorylation following IP with UPF1 or phospho-UPF1 (p-UPF1) specific antibodies. Due to pretreatment of the cells with the PP2a inhibitor okadaic acid, a significant proportion of UPF1 was phosphorylated in these cell extracts, as expected. **(B–G)** qRT-PCR analysis of known cellular NMD-sensitive transcripts *GADD45B*, *PDRG1*, and *MAP3K14* as well as the NMD-insensitive controls *GAPDH*, *RPL32*, and *HPRT1* in the anti-UPF1 IP (relative to the IgG control IP) in EBV^+^ AGS-EBV (B), AKBM (C), P3HR-1 (D), or LCL (E) cells as well as KSHV^+^ HEK293T.rKSHV219 (F) or BCBL1 (G) cells. Data are representative of at least 2 independent experiments and presented as mean ± SD of 3 technical replicates; ns, *p* > 0.05, * *p* ≤ 0.05, *** *p* ≤ 0.001; 1-way ANOVA or 2-sided Student *t* test. The underlying numerical data can be found in S1 Data; original immunoblot can be found in S1 Raw Images. IP, immunoprecipitation; NMD, nonsense-mediated decay; qRT-PCR, quantitative reverse transcription PCR.
(TIF)

S6 Fig NMD controls the abundance of the polycistronic EBV and KSHV transactivator transcripts. **(A)** qRT-PCR analysis of *BRLF1* transcripts in HEK293T cells transfected with 0.5, 1.0, or 1.5 μg of a plasmid encoding the dominant-negative mutant UPF1-R843C, or an empty-vector control, together with a plasmid encoding the complete EBV transactivator locus (left panel, orange) or the *BRLF1* CDS only (right panel, blue) for 24 hours. Data are presented as fold expression relative to the sample without UPF1-R843C (-). **(B)** qRT-PCR analysis of *BZLF1* transcripts in HEK293T cells transfected with increasing amounts of UPF1-R843C as in (A) together with a plasmid encoding the EBV *BZLF1* locus (left panel, orange) or the *BZLF1* CDS (right panel, blue) for 24 hours. Data are presented as fold expression relative to the control sample without UPF1-R843C (-). **(C)** Schematic of the KSHV transactivator locus and the 7 transcripts expressed from this locus. Dashed lines mark spliced introns. CDS, coding sequence. pA, 3′ polyadenylation site. **(D)** qRT-PCR analysis of *Orf50* transcripts in HEK293T cells transfected with 0, 20, 40, or 60 nM si.UPF1 for 24 hours followed by transfection of a plasmid encoding the complete KSHV transactivator locus (left panel, orange) or the *Orf50* CDS (right panel, blue) for 48 hours. Data were normalized to cotransfected *GFP* transcript levels to control for differences in transfection efficiency and presented as fold expression relative to si.Con-transfected cells (-). **(E–G)** Densitometric quantification of the relative signal intensities in 2 or 3 independent experimental replicates of the representative immunoblots presented in Fig 3D (E), 3E (F), and 3H (G). For the highest concentration of si.UPF1 (++++) in panel E, only 1 replicate was included. Data in A, B, and D are representative of at least 3 independent experiments and presented as mean ± SD of 3 biological replicates; ns, *p* > 0.05, * *p* ≤ 0.05, ** *p* ≤ 0.01, *** *p* ≤ 0.001; 1-way ANOVA. The underlying numerical data can be found in S1 Data. EBV, Epstein–Barr virus; GFP, green fluorescent protein; KSHV, Kaposi’s sarcoma-associated herpesvirus; NMD, nonsense-mediated decay; qRT-PCR, quantitative reverse transcription PCR.
(TIF)

S7 Fig Cytotoxicity assessment of NMDI-1 treatment. (**A, B**) Flow cytometry analysis of virus-negative Burkitt lymphoma BJAB (A) or AGS (B) cells treated with 5–100 μM NMDI-1 or DMSO (“0 μM” NMDI-1) for 48 hours and stained with 7-AAD and FITC-Annexin V. Data are presented as mean percentage of live (orange, Annexin V^-^/7-AAD^-^), apoptotic (blue, Annexin V^+^/7-AAD^-^), and necrotic (red, Annexin V^+^/7-AAD^+^) cells for 3 biological replicates. Moreover, 1 μM staurosporine treatment served as positive control for apoptosis/necrosis induction. The underlying numerical data can be found in S1 Data. NMD, nonsense-mediated decay.
(TIF)

S8 Fig GSEA analysis of cellular transcripts enriched with UPF1 in AGS-EBV cells. GSEA was performed by comparing cellular transcripts precipitated with endogneous UPF1 or phospho-UPF1 to those precipitated with the IgG control in untreated or NaB-treated AGS-EBV cells, as described for Figs 2B and S4B–S4D. Analysis of significantly enriched gene sets was performed using the MSigDB and presented as the normalized enrichment scores for the 14 gene sets that were significantly enriched in all 4 conditions (*p*-value < 0.05 and false discovery rate < 0.25). The RNA-seq data have been deposited under NCBI BioProject accession number PRJNA677887. EBV, Epstein–Barr virus; GSEA, Gene Set Enrichment Analysis; MSigDB, Molecular Signatures Database.
(TIF)

S1 Data Raw experimental data of Figs 1–4 and S1, S2 and S5–S7. (XLSX)

S1 Raw Images Raw images of the immunoblots and northern blots presented in Figs 1 and 3 and S2 and S5. (PDF)

## Abbreviations:

ΔΔCt: comparative CT
CDS: coding sequence
DMEM: Dulbecco’s Modified Eagle Medium
EBV: Epstein–Barr virus
EJC: exon junction complex
FBS: fetal bovine serum
GFP: green fluorescent protein
GSEA: Gene Set Enrichment Analysis
HRP: horseradish peroxidase
IgG: Immunoglobulin G
KSHV: Kaposi’s sarcoma-associated herpesvirus
MSigDB: Molecular Signatures Database
NaB: sodium butyrate
NMD: nonsense-mediated decay
nt: nucleotide
PAN RNA: polyadenylated nuclear RNA
p-UPF1: phosphorylated UPF1
PEI: polyethylenimine
PEL: primary effusion lymphoma
PTC: premature termination codon
PVDF: polyvinylidene difluoride
qRT-PCR: quantitative reverse transcription PCR
RFP: red fluorescent protein
RIP-seq: RNA immunoprecipitation sequencing
RNA-seq: RNA sequencing
RPMI: Roswell Park Memorial Institute
RT: room temperature
shRNA: short hairpin RNA
siRNA: small interfering RNA
STAU: Staufen
TSS: transcription start site
WT: wild-type
