# Supplementary figures and images for: Nonsense-mediated decay controls the reactivation of the oncogenic herpesviruses EBV and KSHV
Source: PLoS Biol. 2021 Feb 17;19(2):e3001097. doi: 10.1371/journal.pbio.3001097 (PMC7888593; doi:10.1371/journal.pbio.3001097)

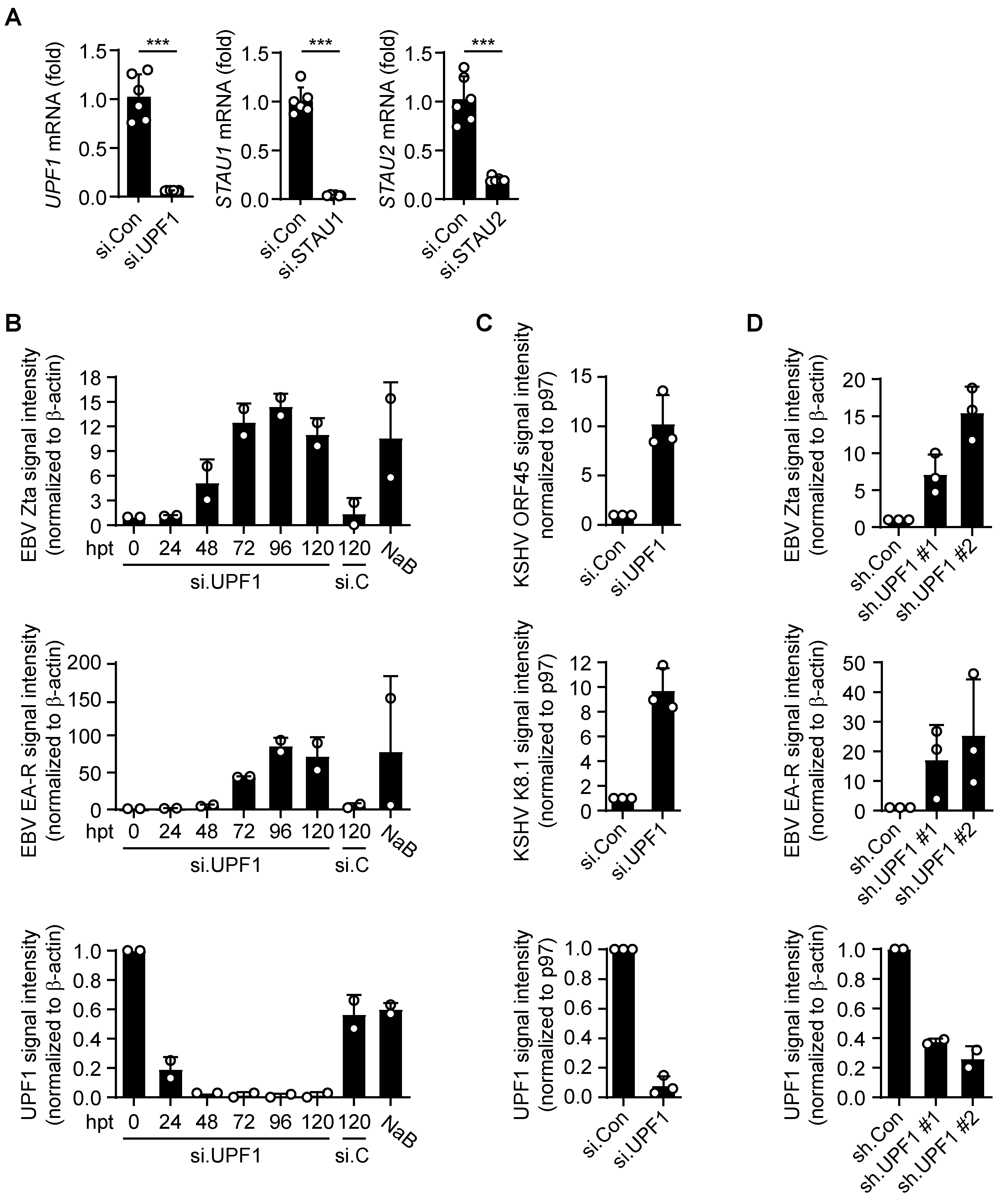

Supplement: S1 Fig — (A) Representative qRT-PCR analysis of UPF1, STAU1, and STAU2 knockdown efficiency in AGS-EBV cells transfected with the indicated siRNAs for 96 hours, displayed as fold expression relative to si.Con-transfected cells. (B–D) Densitometric quantification of the relative signal intensities in 2 or 3 independent biological replicates of the representative immunoblots presented in Fig 1G (B), 1H (C), and 1J (D). The underlying numerical data can be found in S1 Data. EBV, Epstein–Barr virus; qRT-PCR, quantitative reverse transcription PCR; siRNA, small interfering RNA; STAU, Staufen. (TIF) [file pbio.3001097.s001.tif]

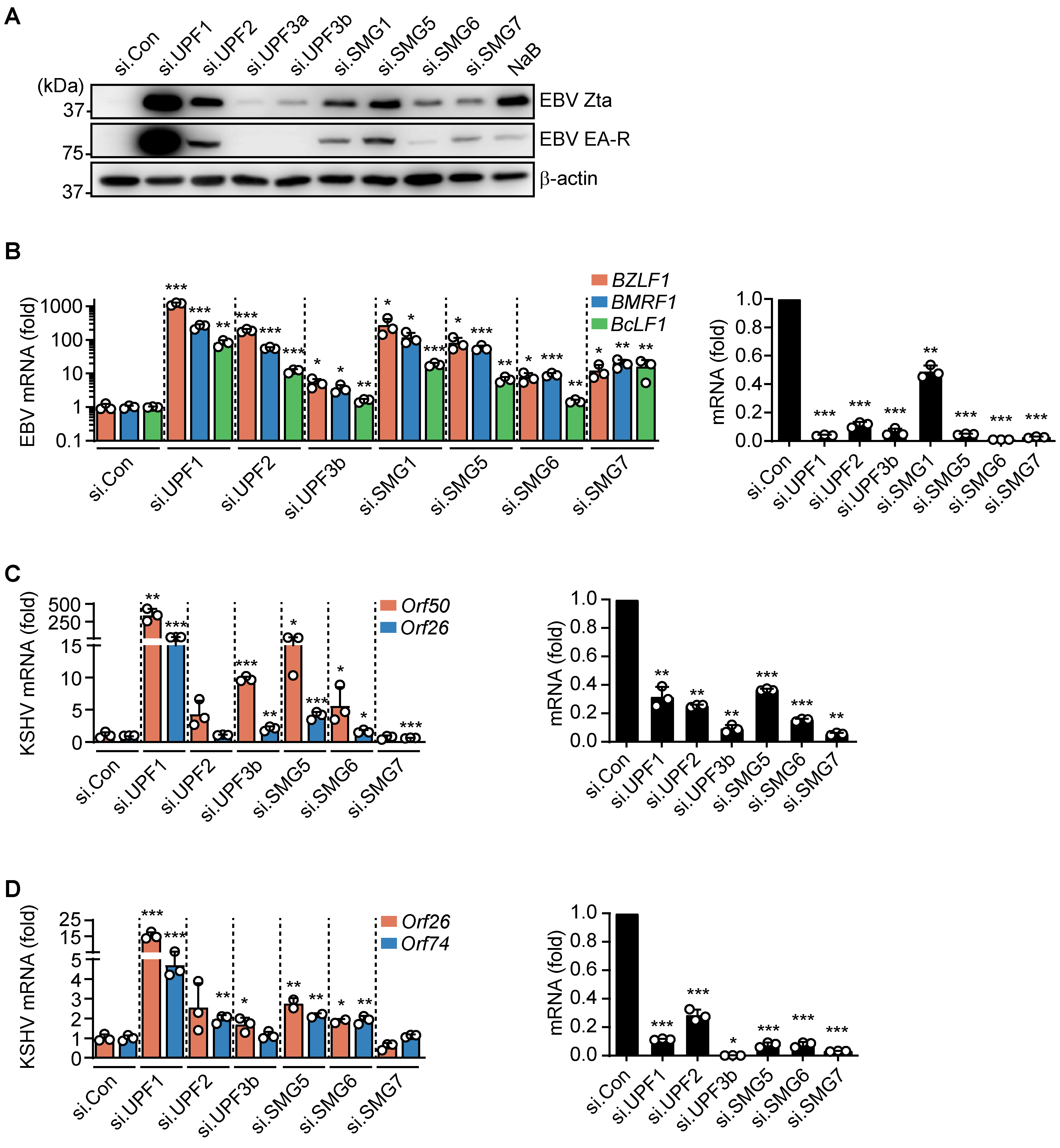

Supplement: S2 Fig — (A) Immunoblotting analysis of EBV proteins Zta and EA-R in AGS-EBV cells transfected for 96 hours with siRNAs targeting the indicated NMD factors, or a nontargeting control siRNA (si.Con). β-actin served as loading control. Treatment with 2.5 mM sodium butyrate (NaB) for 24 hours served as positive control for reactivation. (B) qRT-PCR analysis of EBV lytic genes BZLF1, BMRF1, and BcLF1 (left panel) and silencing efficiency of the respective NMD components (right panel) in AGS-EBV cells treated as in (A), presented as fold expression relative to si.Con. (C) qRT-PCR analysis of KSHV Orf50 and Orf26 transcripts (left panel) and knockdown efficiency of the respective NMD components (right panel) in HEK293T.rKSHV219 cells transfected with siRNAs targeting the indicated NMD genes for 72 hours, presented as fold expression relative to si.Con. (D) qRT-PCR analysis of KSHV lytic Orf26 and Orf74 transcripts (left panel) and knockdown efficiency of the respective NMD components (right panel) in iSLK.rKSHV219 cells transfected with the indicated siRNAs for 96 hours, presented as fold expression relative to si.Con. Of note, SMG1 could not be efficiently silenced in HEK293T.rKSHV219 or iSLK.rKSHV219 cells and therefore was not tested. Data are presented as mean ± SD of at least 3 biological replicates; * p ≤ 0.05, ** p ≤ 0.01, *** p ≤ 0.001. The underlying numerical data can be found in S1 Data; original immunoblots can be found in S1 Raw Images. EBV, Epstein–Barr virus; KSHV, Kaposi’s sarcoma-associated herpesvirus; NMD, nonsense-mediated decay; qRT-PCR, quantitative reverse transcription PCR; siRNA, small interfering RNA. (TIF) [file pbio.3001097.s002.tif]

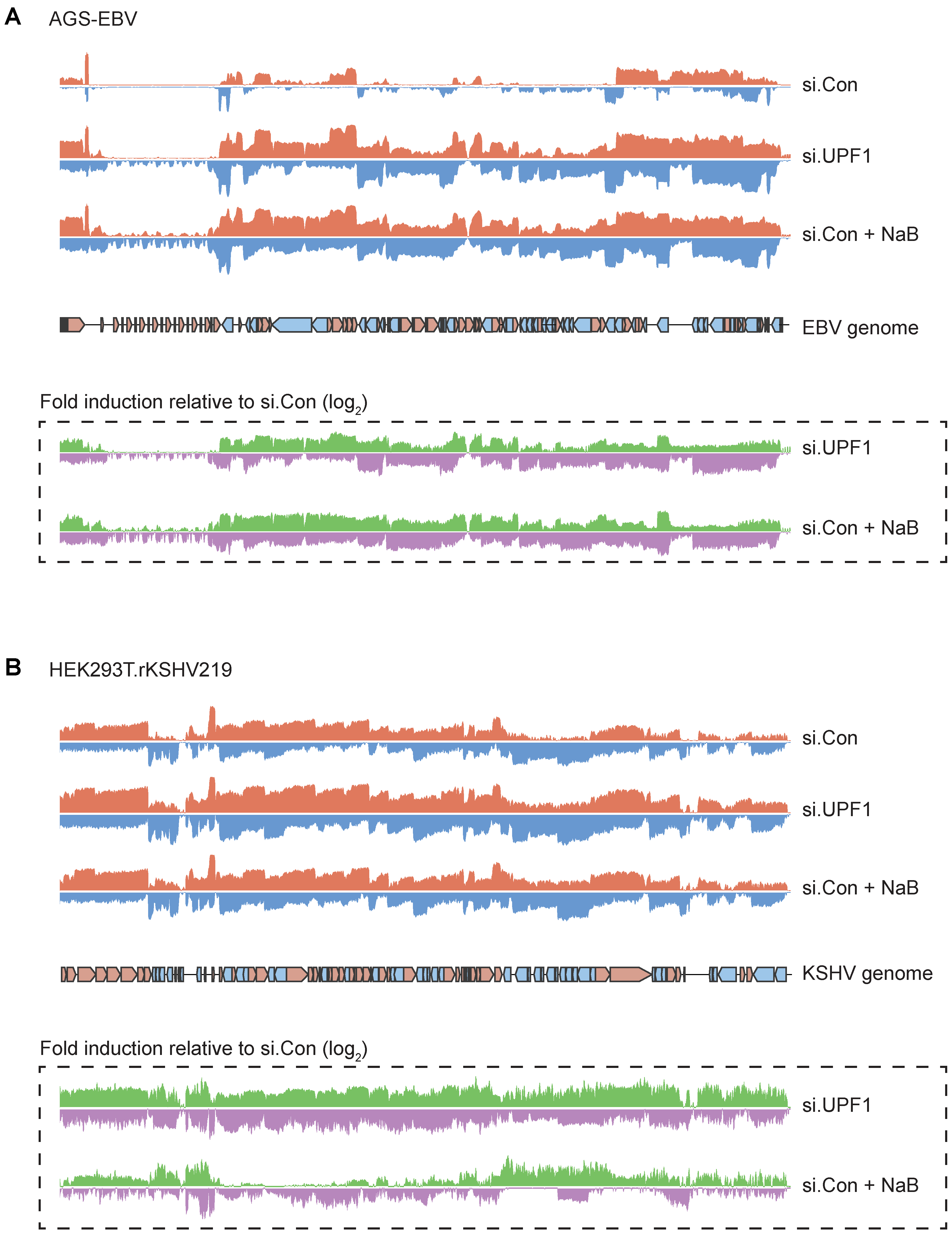

Supplement: S3 Fig — (A) Whole genome RNA-seq coverage plots (log2 scale) of RNA-seq reads mapping to the forward (orange) or reverse (blue) strands of the EBV genome. AGS-EBV cells were transfected for 96 hours with UPF1-specific siRNAs (si.UPF1) or nontargeting control siRNAs (si.Con) with or without treatment with 2.5 mM NaB for the last 24 hours, followed by RNA purification and whole transcriptome RNA-seq analysis. Plots are aligned to a schematic representation of the EBV genome depicting annotated ORFs on the forward (orange) and reverse (blue) strands. Lower panel displays the fold induction of mapped reads of the si.UPF1 and NaB-treated samples relative to si.Con sample (log2 scale). (B) Whole genome RNA-seq coverage plots (log2 scale) of RNA-seq reads mapping to the forward (orange) or reverse (blue) strands of the KSHV genome, derived from HEK293T.rKSHV219 cell extracts treated and displayed as in (A). The RNA-seq data have been deposited under NCBI BioProject accession number PRJNA677887. EBV, Epstein–Barr virus; KSHV, Kaposi’s sarcoma-associated herpesvirus; RNA-seq, RNA sequencing. (TIF) [file pbio.3001097.s003.tif]

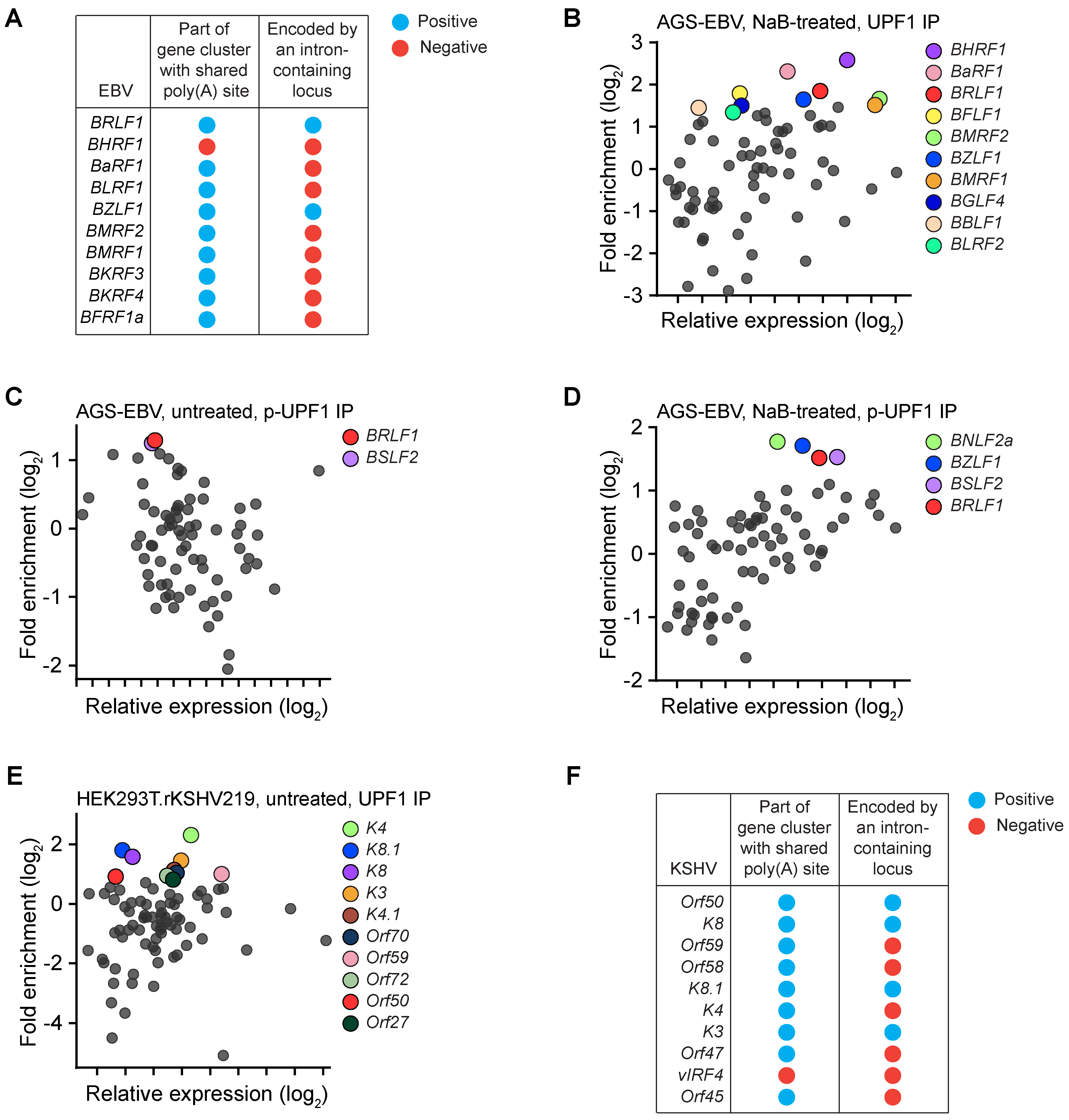

Supplement: S4 Fig — (A) Overview of the 10 EBV transcripts presented in Fig 2B that were found to be significantly enriched with endogenous UPF1 in AGS-EBV cells indicating whether they are (blue circle) or are not (red circle) encoded in a viral gene cluster with a shared polyadenylation (poly(A)) site and/or derived from an intron-containing locus. (B) Enrichment of UPF1-associated EBV transcripts following UPF1 IP from AGS-EBV cells treated with 2.5 mM NaB for 24 hours, as determined by RNA-seq analysis and presented as fold enrichment in the UPF1 IP relative to the IgG control IP (log2 values; y-axis) versus relative abundance in the total input RNA (log2 values; x-axis). Labels in plot indicate the 10 viral transcripts with the greatest enrichment. (C, D) Enrichment of UPF1-associated EBV transcripts following phospho-UPF1 IP from untreated AGS-EBV cells (C) or AGS-EBV cells treated with 2.5 mM NaB for 24 hours (D), determined by RNA-seq and presented as in (B). Labels in plots indicate the significantly enriched EBV transcripts. (E) KSHV transcript enrichment with UPF1 following UPF1 IP from HEK293T.rKSHV219 cell extracts, determined by RNA-seq and presented as in (B). Labels in plot indicate the 10 viral transcripts with the greatest enrichment. (F) Overview of the 10 KSHV transcripts presented in Fig 2C that were found to be highly enriched with endogenous UPF1 in BCBL1 cells indicating whether they are (blue circle) or are not (red circle) encoded in viral gene cluster with a shared polyadenylation (poly(A)) site and/or derived from an intron-containing locus. The RNA-seq data have been deposited under NCBI BioProject accession number PRJNA677887. EBV, Epstein–Barr virus; IP, immunoprecipitation; KSHV, Kaposi’s sarcoma-associated herpesvirus; RIP-seq, RNA immunoprecipitation sequencing; RNA-seq, RNA sequencing. (TIF) [file pbio.3001097.s004.tif]

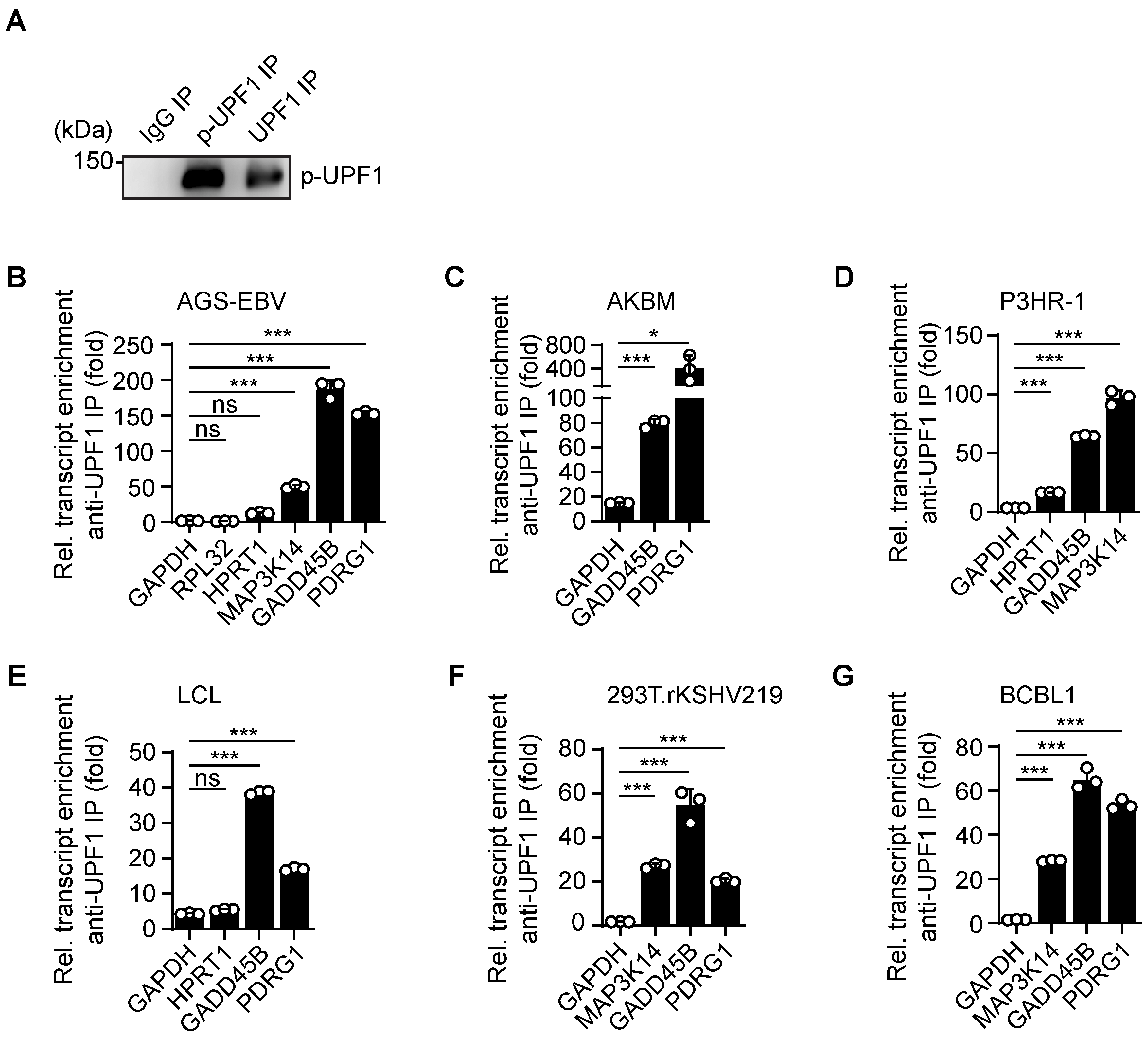

Supplement: S5 Fig — (A) Representative immunoblotting analysis showing UPF1 phosphorylation following IP with UPF1 or phospho-UPF1 (p-UPF1) specific antibodies. Due to pretreatment of the cells with the PP2a inhibitor okadaic acid, a significant proportion of UPF1 was phosphorylated in these cell extracts, as expected. (B–G) qRT-PCR analysis of known cellular NMD-sensitive transcripts GADD45B, PDRG1, and MAP3K14 as well as the NMD-insensitive controls GAPDH, RPL32, and HPRT1 in the anti-UPF1 IP (relative to the IgG control IP) in EBV+ AGS-EBV (B), AKBM (C), P3HR-1 (D), or LCL (E) cells as well as KSHV+ HEK293T.rKSHV219 (F) or BCBL1 (G) cells. Data are representative of at least 2 independent experiments and presented as mean ± SD of 3 technical replicates; ns, p > 0.05, * p ≤ 0.05, *** p ≤ 0.001; 1-way ANOVA or 2-sided Student t test. The underlying numerical data can be found in S1 Data; original immunoblot can be found in S1 Raw Images. IP, immunoprecipitation; NMD, nonsense-mediated decay; qRT-PCR, quantitative reverse transcription PCR. (TIF) [file pbio.3001097.s005.tif]

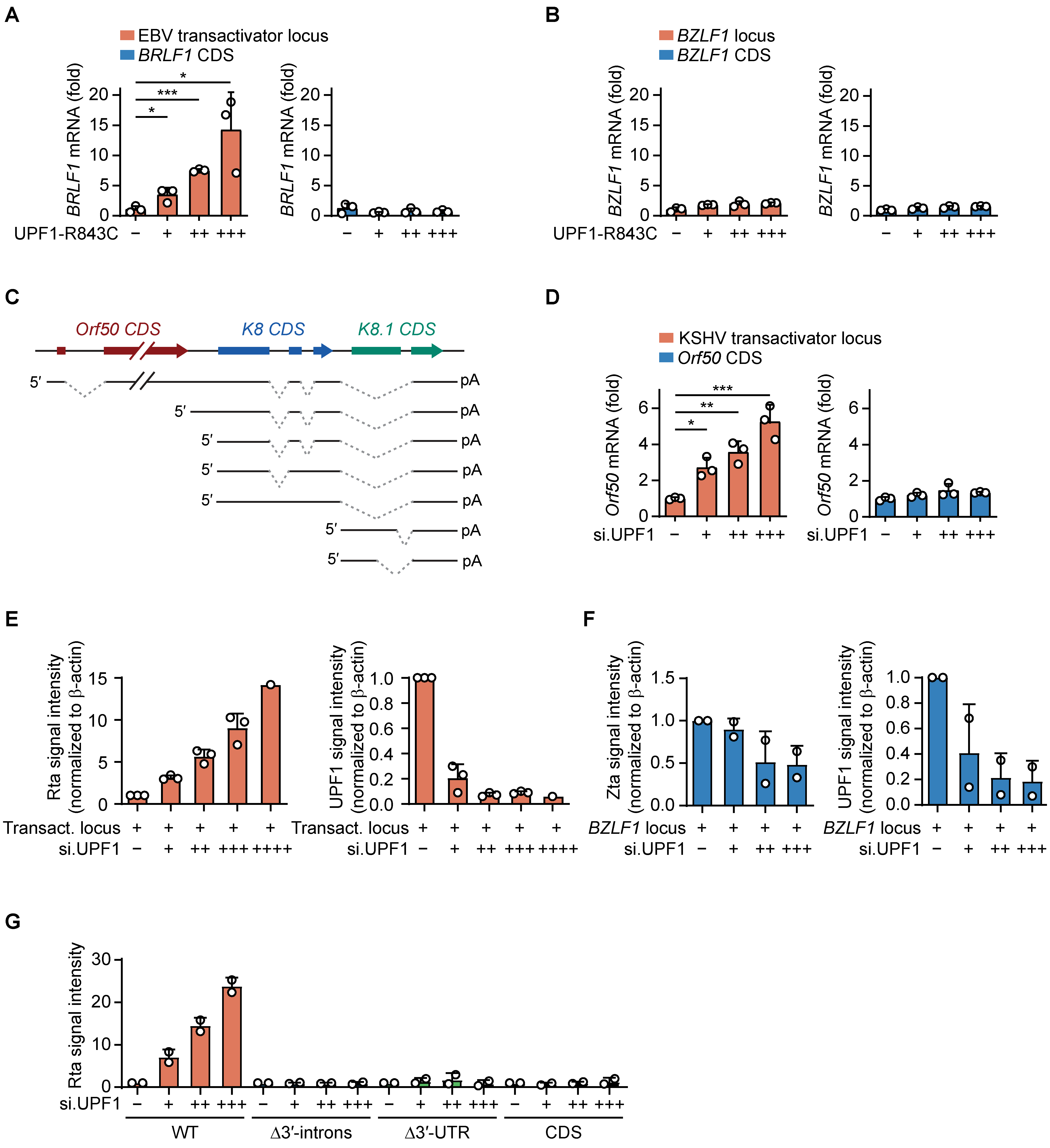

Supplement: S6 Fig — (A) qRT-PCR analysis of BRLF1 transcripts in HEK293T cells transfected with 0.5, 1.0, or 1.5 μg of a plasmid encoding the dominant-negative mutant UPF1-R843C, or an empty-vector control, together with a plasmid encoding the complete EBV transactivator locus (left panel, orange) or the BRLF1 CDS only (right panel, blue) for 24 hours. Data are presented as fold expression relative to the sample without UPF1-R843C (-). (B) qRT-PCR analysis of BZLF1 transcripts in HEK293T cells transfected with increasing amounts of UPF1-R843C as in (A) together with a plasmid encoding the EBV BZLF1 locus (left panel, orange) or the BZLF1 CDS (right panel, blue) for 24 hours. Data are presented as fold expression relative to the control sample without UPF1-R843C (-). (C) Schematic of the KSHV transactivator locus and the 7 transcripts expressed from this locus. Dashed lines mark spliced introns. CDS, coding sequence. pA, 3′ polyadenylation site. (D) qRT-PCR analysis of Orf50 transcripts in HEK293T cells transfected with 0, 20, 40, or 60 nM si.UPF1 for 24 hours followed by transfection of a plasmid encoding the complete KSHV transactivator locus (left panel, orange) or the Orf50 CDS (right panel, blue) for 48 hours. Data were normalized to cotransfected GFP transcript levels to control for differences in transfection efficiency and presented as fold expression relative to si.Con-transfected cells (-). (E–G) Densitometric quantification of the relative signal intensities in 2 or 3 independent experimental replicates of the representative immunoblots presented in Fig 3D (E), 3E (F), and 3H (G). For the highest concentration of si.UPF1 (++++) in panel E, only 1 replicate was included. Data in A, B, and D are representative of at least 3 independent experiments and presented as mean ± SD of 3 biological replicates; ns, p > 0.05, * p ≤ 0.05, ** p ≤ 0.01, *** p ≤ 0.001; 1-way ANOVA. The underlying numerical data can be found in S1 Data. EBV, Epstein–Barr virus; GFP, green fluorescent protein; [file pbio.3001097.s006.tif]

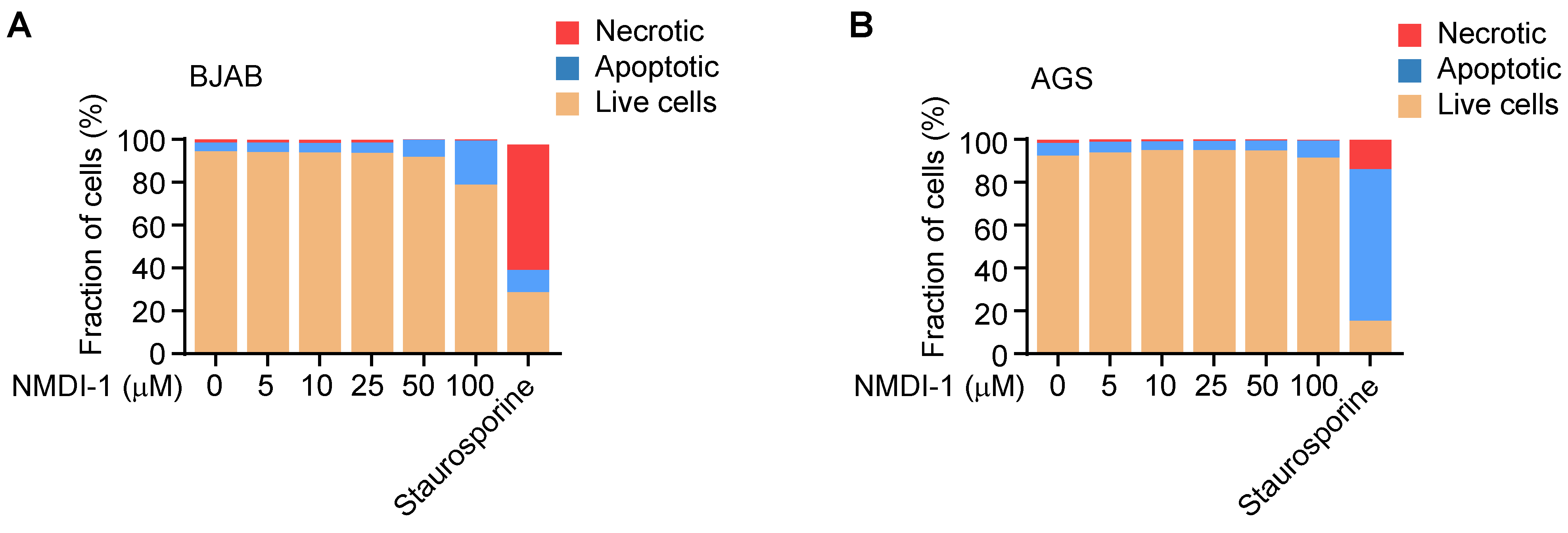

Supplement: S7 Fig — (A, B) Flow cytometry analysis of virus-negative Burkitt lymphoma BJAB (A) or AGS (B) cells treated with 5–100 μM NMDI-1 or DMSO (“0 μM” NMDI-1) for 48 hours and stained with 7-AAD and FITC-Annexin V. Data are presented as mean percentage of live (orange, Annexin V-/7-AAD-), apoptotic (blue, Annexin V+/7-AAD-), and necrotic (red, Annexin V+/7-AAD+) cells for 3 biological replicates. Moreover, 1 μM staurosporine treatment served as positive control for apoptosis/necrosis induction. The underlying numerical data can be found in S1 Data. NMD, nonsense-mediated decay. (TIF) [file pbio.3001097.s007.tif]

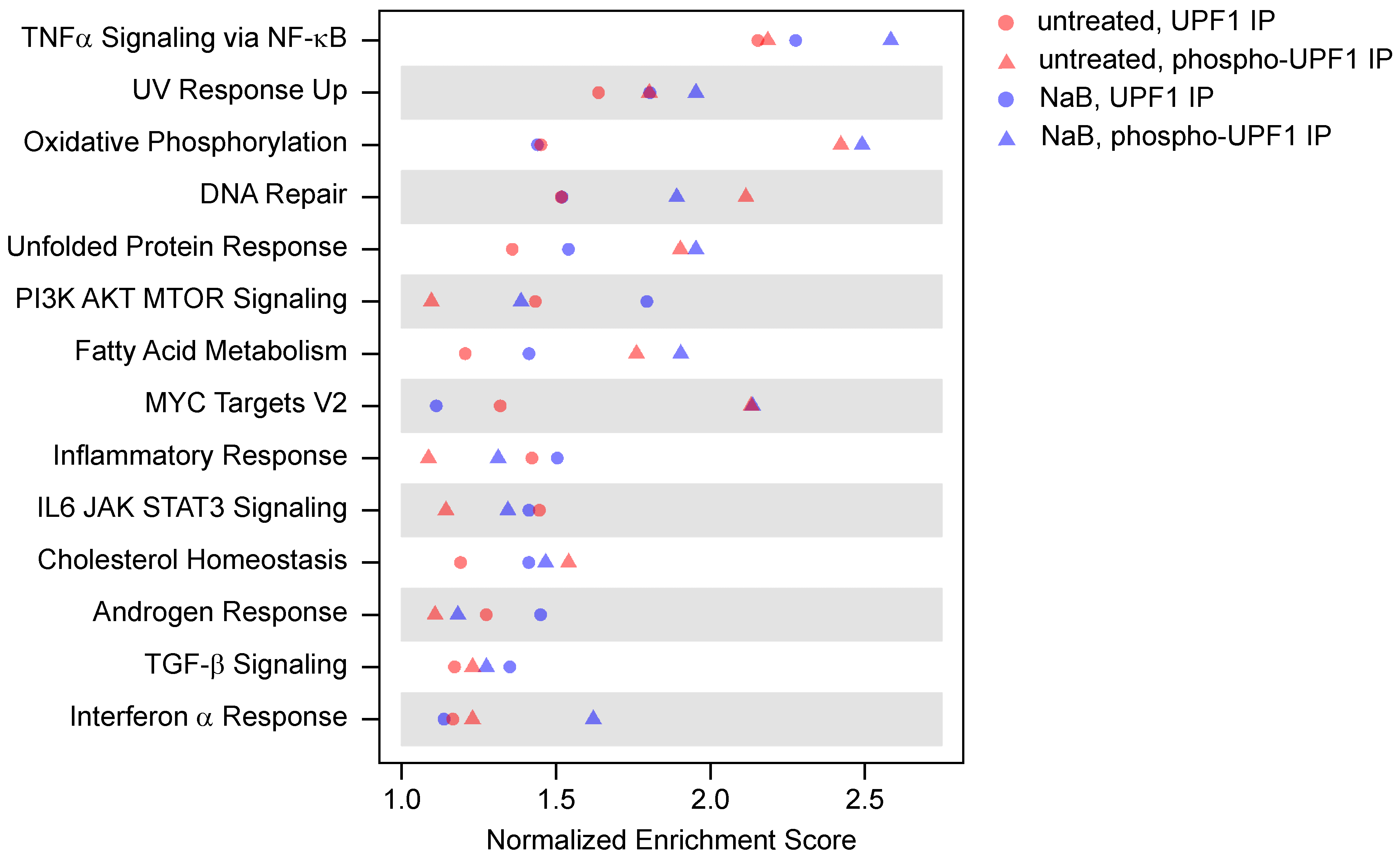

Supplement: S8 Fig — GSEA was performed by comparing cellular transcripts precipitated with endogneous UPF1 or phospho-UPF1 to those precipitated with the IgG control in untreated or NaB-treated AGS-EBV cells, as described for Figs 2B and S4B–S4D. Analysis of significantly enriched gene sets was performed using the MSigDB and presented as the normalized enrichment scores for the 14 gene sets that were significantly enriched in all 4 conditions (p-value < 0.05 and false discovery rate < 0.25). The RNA-seq data have been deposited under NCBI BioProject accession number PRJNA677887. EBV, Epstein–Barr virus; GSEA, Gene Set Enrichment Analysis; MSigDB, Molecular Signatures Database. (TIF) [file pbio.3001097.s008.tif]
