# Supplementary material for: Nonsense-mediated decay controls the reactivation of the oncogenic herpesviruses EBV and KSHV
Source: PLoS Biol. 2021 Feb 17;19(2):e3001097. doi: 10.1371/journal.pbio.3001097 (PMC7888593; doi:10.1371/journal.pbio.3001097)

Raw immunoblotting and Northern blotting images

Figure 1G

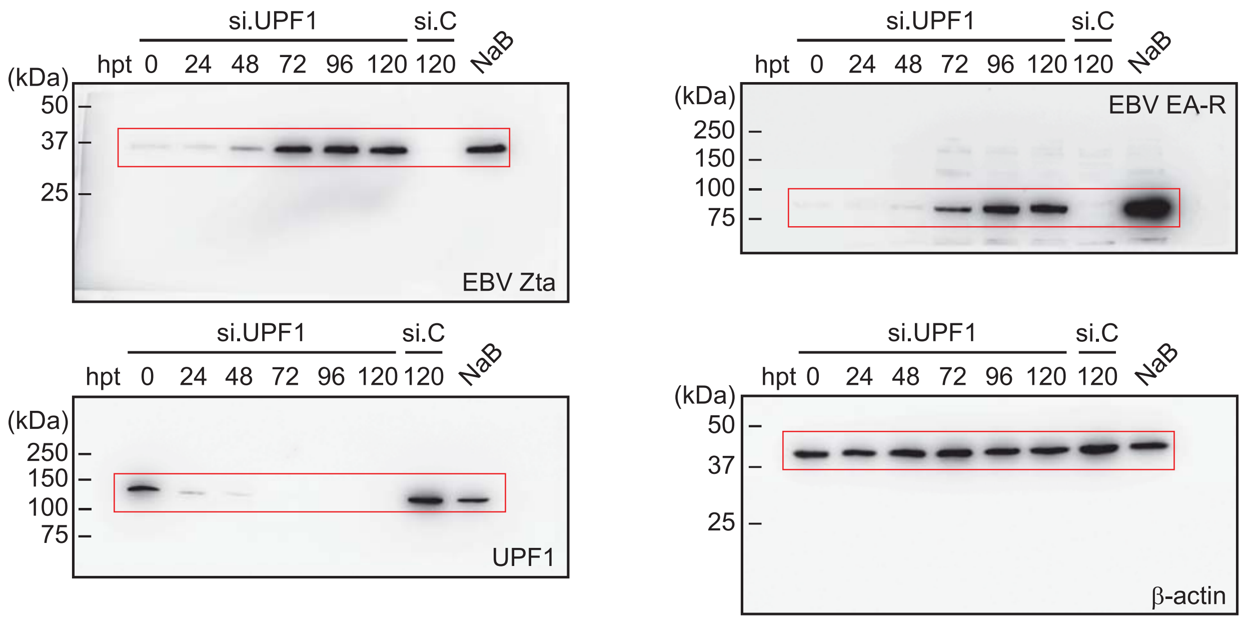

Figure 1H

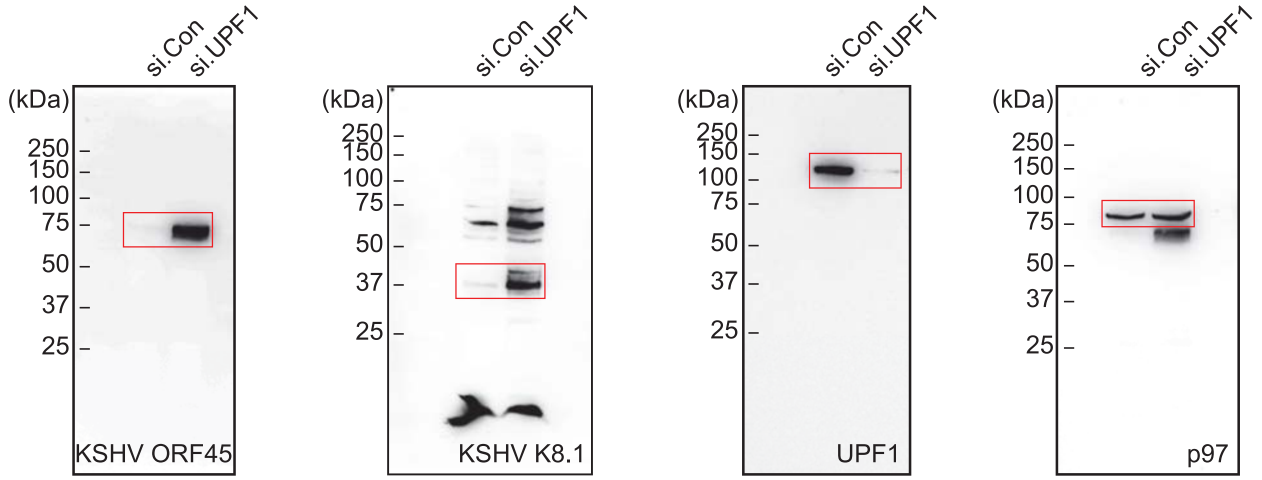

Figure 1J

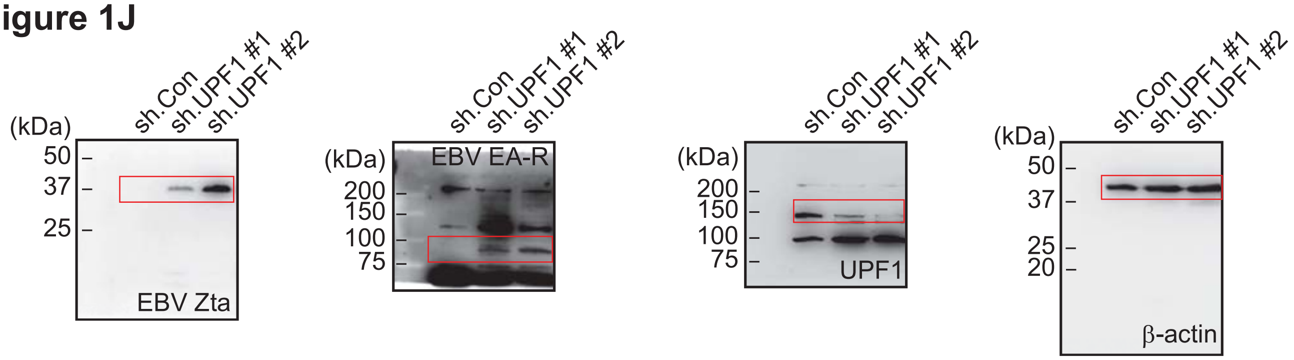

Figure 3D

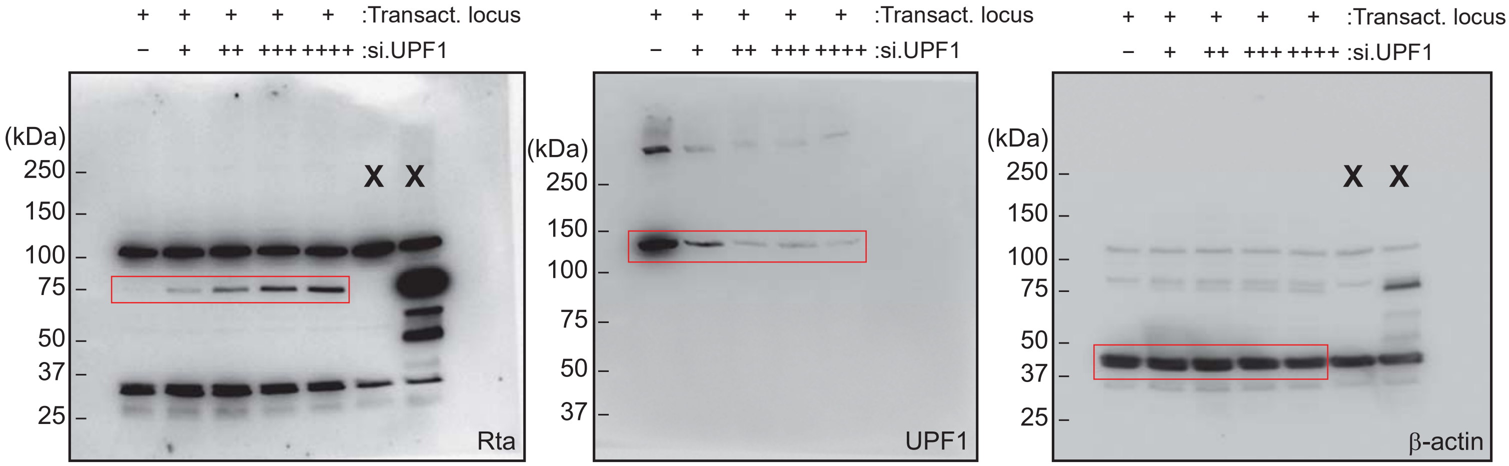

Figure 3E

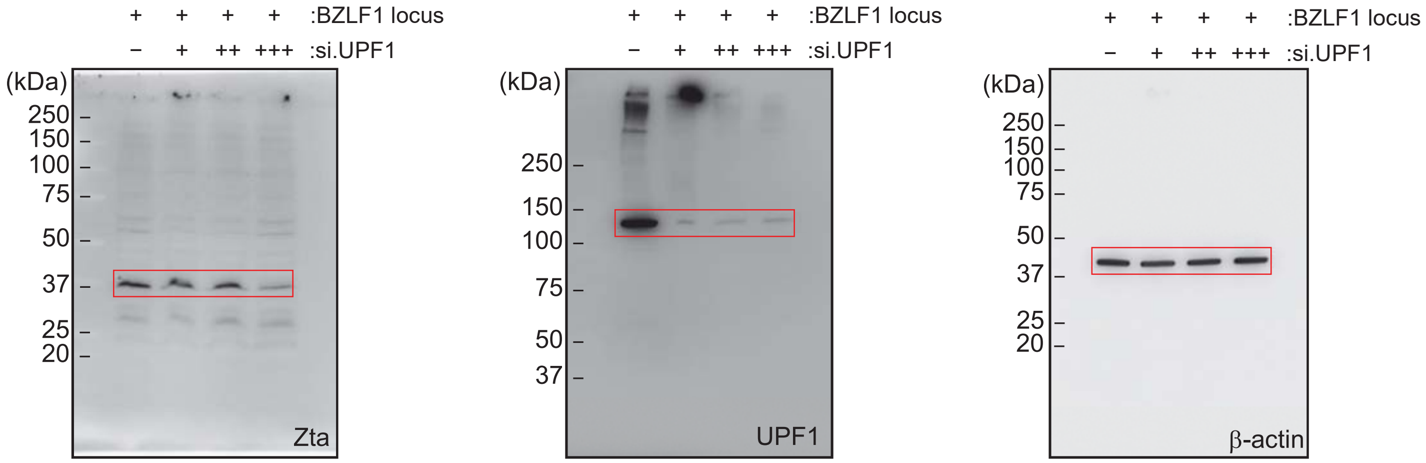

Figure 3F

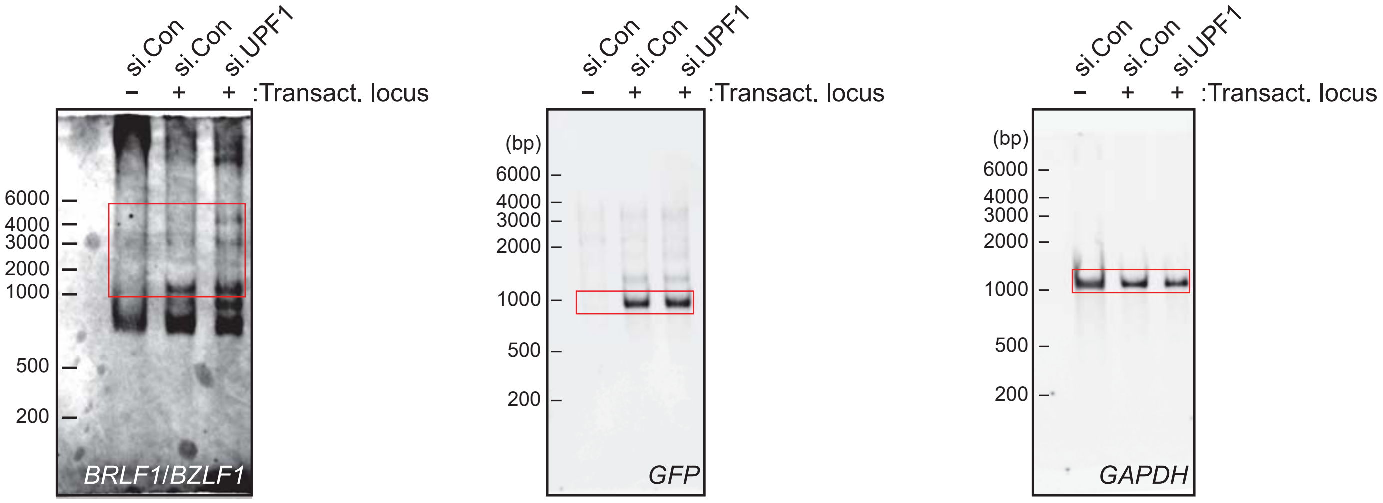

Figure 3H

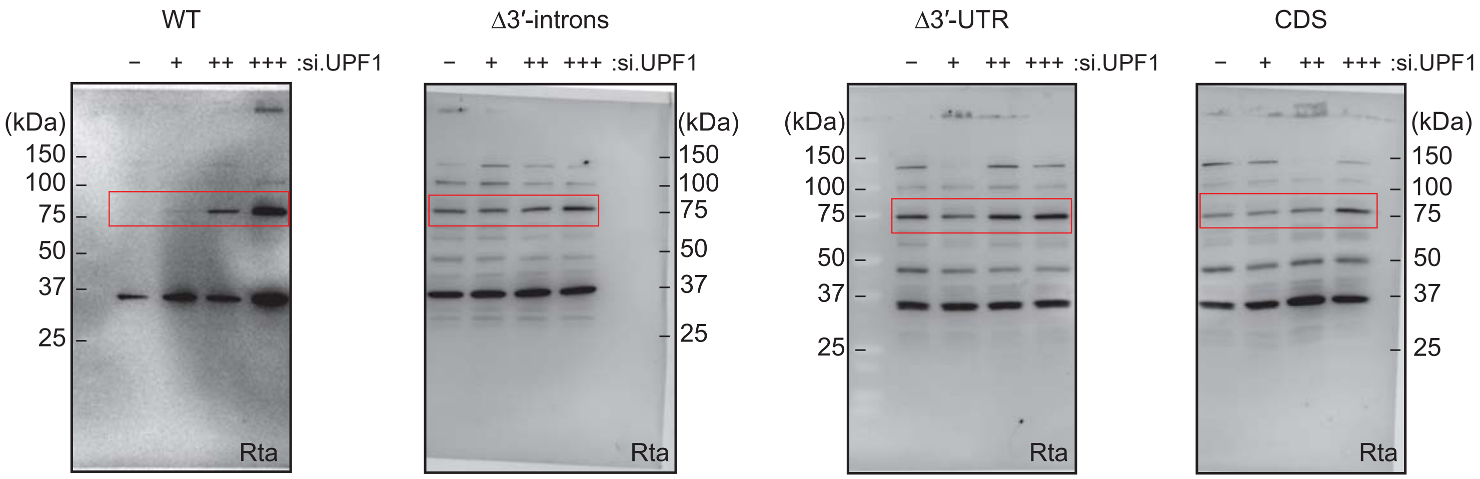

Figure 3I

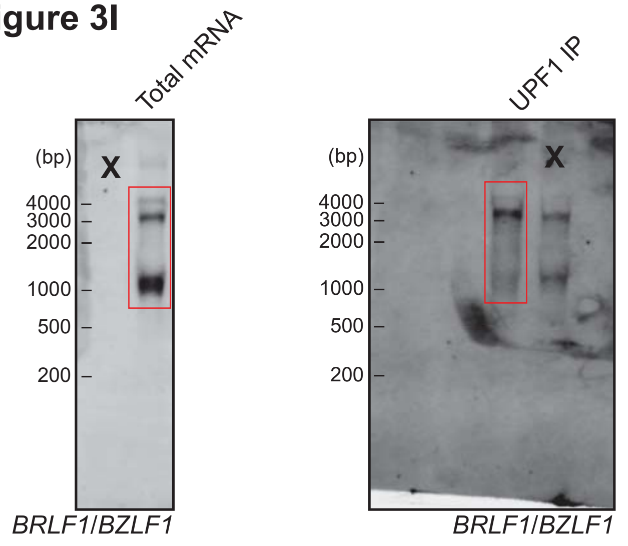

Supporting Figure 2A

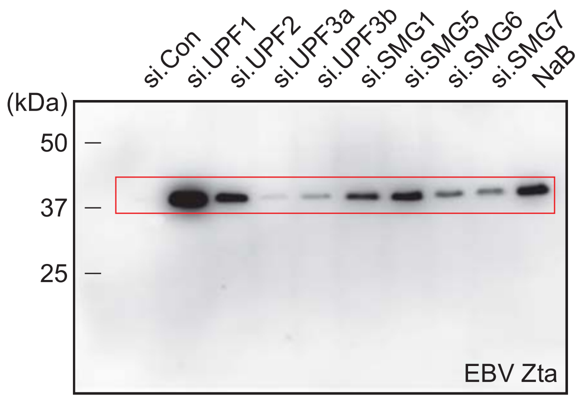

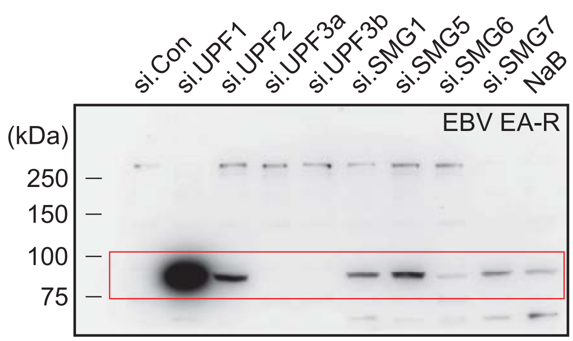

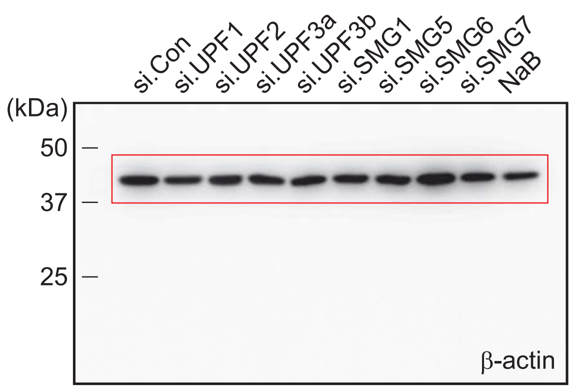

Supporting Figure 5A

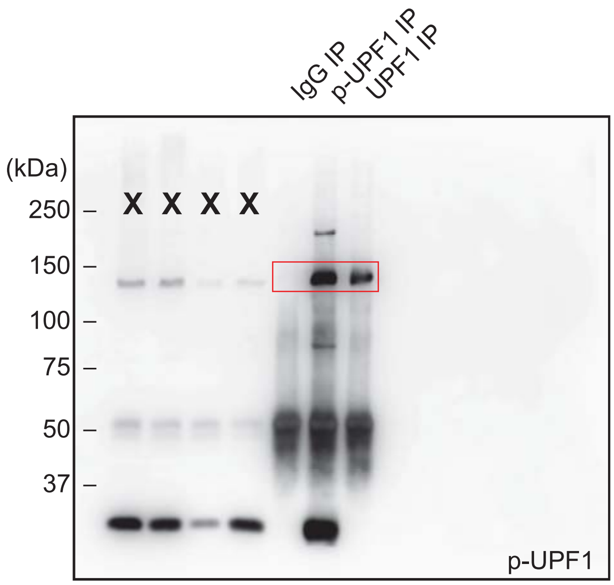

Supplement: S1 Raw Images — (PDF) [file pbio.3001097.s010.pdf]
